# Supplementary material for: Phenotypic variability and trait-specific selection in Aegle marmelos Correa genotypes based on morphological and quality traits
Source: PLoS One. 2026 Apr 20;21(4):e0347746. doi: 10.1371/journal.pone.0347746 (PMC13095130; doi:10.1371/journal.pone.0347746)
Supplement: Attachment — Submitted filename: PONE-D-26-04072 reviewed.pdf [file pone.0347746.s002.pdf]

PLOS One

Multivariate analyses of morphological and chemical variations in Aegle marmelos Correa genotypes

--Manuscript Draft--

**Manuscript Number:**	PONE-D-26-04072
**Article Type:**	Research Article
**Full Title:**	Multivariate analyses of morphological and chemical variations in Aegle marmelos Correa genotypes
**Short Title:**	Morphological and Chemical Diversity in Aegle marmelos Genotypes
**Corresponding Author:**	Ali Khadivi<br>Arak University<br>Arak, IRAN, ISLAMIC REPUBLIC OF
**Keywords:**	Aegle marmelos, Genetic diversity, Heritability, GCV, PCA, Cluster analysis
**Abstract:**	This study aimed to assess the genetic variability in Aegle marmelos Correa to develop trait-specific genotypes based on morphological and qualitative traits. The evaluation focused on both morphological and qualitative characteristics within the gene pool of this species. High phenotypic coefficient of variation (PCV) and genotypic coefficient of variation (GCV) were observed for traits such as shell weight, fruit weight, and pulp weight, indicating substantial genetic diversity and strong potential for selective breeding within the germplasm. Heritability estimates ranged widely, with fruit weight showing a low 0.07% and shell weight a high 92.23%, reflecting the significant impact of environmental factors on trait expression. Principal Component Analysis (PCA) revealed that the first principal component (PC1) explained 40.19% of the total variation, with an eigenvalue of 8.12. The first six principal components collectively accounted for 80.77% of total variability. Genotypes CHESB-25 and CHESB-29 exhibited the highest positive PC scores for PC1 and PC2, identifying them as superior selections. Cluster analysis identified six distinct clusters of genotypes, with Cluster V being the largest and Cluster VI the smallest. This clustering highlights the genetic diversity among the bael genotypes and provides a basis for breeding and selection strategies. Cluster IV emerged as the most promising, consistently showing the highest values for key attributes such as shell weight, fruit weight, and fruit yield per plant. Therefore, prioritizing Cluster IV is recommended for selecting superior varieties and developing new cultivars. The study also noted that fruit yield per plant positively correlated with traits like shell weight and fruit weight, emphasizing the importance of these traits for yield improvement. Conversely, negative correlations with seed percent, shell percent, and phenolic content suggest these traits may be less beneficial for enhancing yield. The hierarchical clustering heat map of the 101 bael germplasms offers a detailed perspective on the relationships between various traits and germplasms. The results offer vital information for creating A. marmelos cultivars with higher yields and better quality. For breeding programs, targeted selection is made possible by the discovery of important clusters and superior genotypes (CHESB-25 and CHESB-29). Given the high level of genetic variation found, hybridization may be able to improve desired characteristics like fruit output and weight. Overall, the findings offer important insights for selecting elite genotypes and advancing breeding programs.
**Order of Authors:**	A.K. Singh
	Vikas Yadav
	Lalu Prasad Yadav
	K. Gangadhara
	Anil Pawar
	Jagadish Rane
	P. Ravat
	A. Sahil

Powered by Editorial Manager® and ProduXion Manager® from Aries Systems Corporation

	Prashant Kaushik
	Ali Khadivi
	Yazgan Tunç
**Additional Information:**	
**Question**	**Response**
**Financial Disclosure**<br><br>Enter a financial disclosure statement that describes the sources of funding for the work included in this submission. Review the submission guidelines for detailed requirements. View published research articles from *PLOS ONE* for specific examples.<br><br>This statement is required for submission and **will appear in the published article** if the submission is accepted. Please make sure it is accurate.<br><br>**Funded studies**<br>Enter a statement with the following details:<br>• Initials of the authors who received each award<br>• Grant numbers awarded to each author<br>• The full name of each funder<br>• URL of each funder website<br>• Did the sponsors or funders play any role in the study design, data collection and analysis, decision to publish, or preparation of the manuscript?<br><br>Did you receive funding for this work?	The author(s) received no specific funding for this work.
**Competing Interests**<br><br>Use the instructions below to enter a competing interest statement for this submission. On behalf of all authors, disclose any competing interests that could be perceived to bias this work—acknowledging all financial support and any other relevant financial or non-financial competing interests.<br><br>This statement is required for submission	The authors have declared that no competing interests exist.

and **will appear in the published article** if the submission is accepted. Please make sure it is accurate and that any funding sources listed in your Funding Information later in the submission form are also declared in your Financial Disclosure statement.<br><br>View published research articles from *PLOS ONE* for specific examples.<br><br>**NO authors have competing interests**<br><br>Enter: *The authors have declared that no competing interests exist.*<br><br>**Authors with competing interests**<br><br>Enter competing interest details beginning with this statement:<br><br>*I have read the journal's policy and the authors of this manuscript have the following competing interests: [insert competing interests here]*<br><br>* typeset	
**Ethics Statement**<br><br>Enter an ethics statement for this submission. This statement is required if the study involved:<br><br>• Human participants<br>• Human specimens or tissue<br>• Vertebrate animals or cephalopods<br>• Vertebrate embryos or tissues<br>• Field research<br><br>Write "N/A" if the submission does not require an ethics statement.<br><br>General guidance is provided below. Consult the submission guidelines for detailed instructions. **Make sure that all information entered here is included in the Methods section of the manuscript.**	N/A

**Format for specific study types**<br><br>**Human Subject Research (involving human participants and/or tissue)**<br>• Give the name of the institutional review board or ethics committee that approved the study<br>• Include the approval number and/or a statement indicating approval of this research<br>• Indicate the form of consent obtained (written/oral) or the reason that consent was not obtained (e.g. the data were analyzed anonymously)<br><br>**Animal Research (involving vertebrate animals, embryos or tissues)**<br>• Provide the name of the Institutional Animal Care and Use Committee (IACUC) or other relevant ethics board that reviewed the study protocol, and indicate whether they approved this research or granted a formal waiver of ethical approval<br>• Include an approval number if one was obtained<br>• If the study involved *non-human primates*, add *additional details* about animal welfare and steps taken to ameliorate suffering<br>• If anesthesia, euthanasia, or any kind of animal sacrifice is part of the study, include briefly which substances and/or methods were applied<br><br>**Field Research**<br><br>Include the following details if this study involves the collection of plant, animal, or other materials from a natural setting:<br>• Field permit number<br>• Name of the institution or relevant body that granted permission	
**Data Availability**<br><br>Authors are required to make all data underlying the findings described fully available, without restriction, and from the time of publication. PLOS allows rare exceptions to address legal and ethical concerns. See the PLOS Data Policy and FAQ for detailed information.	Yes - all data are fully available without restriction

A Data Availability Statement describing where the data can be found is required at submission. Your answers to this question constitute the Data Availability Statement and **will be published in the article**, if accepted.<br><br>**Important:** Stating 'data available on request from the author' is not sufficient. If your data are only available upon request, select 'No' for the first question and explain your exceptional situation in the text box.<br><br>Do the authors confirm that all data underlying the findings described in their manuscript are fully available without restriction?	
**Describe where the data may be found in full sentences. If you are copying our sample text, replace any instances of XXX with the appropriate details.**<br><br>• If the data are **held or will be held in a public repository**, include URLs, accession numbers or DOIs. If this information will only be available after acceptance, indicate this by ticking the box below. For example: *All XXX files are available from the XXX database (accession number(s) XXX, XXX.*).<br>• If the data are all contained **within the manuscript and/or Supporting Information files**, enter the following: *All relevant data are within the manuscript and its Supporting Information files.*<br>• If neither of these applies but you are able to provide **details of access elsewhere**, with or without limitations, please do so. For example:<br><br>*Data cannot be shared publicly because of [XXX]. Data are available from the XXX Institutional Data Access / Ethics Committee (contact via XXX) for researchers who meet the criteria for access to confidential data.*<br><br>*The data underlying the results presented in the study are available from (include the name of the third party*	All relevant data are within the manuscript and its Supporting Information files.

and contact information or URL).<br>• This text is appropriate if the data are owned by a third party and authors do not have permission to share the data.<br><br>* typeset	
Additional data availability information:	

Powered by Editorial Manager® and ProduXion Manager® from Aries Systems Corporation

Manuscript file Click here to access/download;Manuscript;Full MS.docx

Multivariate analyses of morphological and chemical variations in *Aegle marmelos* Correa genotypes

A.K. Singh[1], Vikas Yadav[1], Lalu Prasad Yadav[1*], K. Gangadhara[1], Anil Pawar[1], Jagadish Rane[2], P. Ravat[1], A. Sahil[1], Prashant Kaushik[3], Ali Khadivi[4*], Yazgan Tunç[5]

[1]ICAR- Central Horticultural Experiment Station (CIAH RS,) Godhra - 389340, Gujarat, India

[2]ICAR-Central Institute for Arid Horticulture, Beechwal- 334006, Bikaner, Rajasthan, India

[3]Chaudhary Charan Singh Haryana Agricultural University, Hisar - 125 004, Haryana, India

[4]Department of Horticultural Sciences, Faculty of Agriculture and Natural Resources, Arak University, 38156-8-8349 Arak, Iran

[5]Republic of Türkiye, Ministry of Agriculture and Forestry, General Directorate of Agricultural Research and Policies, Hatay Olive Research Institute Directorate, Hassa Station, 31700, Hassa, Hatay, Türkiye

Corresponding authors: Lalu Prasad Yadav (yadavlaluprasad682@gmail.com) and Ali Khadivi (a-khadivi@araku.ac.ir)

ORCID ID: Vikas Yadav (0000-0002-0786-0375), Lalu Prasad Yadav (0000-0002-0932-5990), Jagadish Rane (0000-0001-8238-0219), Yazgan Tunç (0000-0002-3228-8657), Ali Khadivi (0000-0001-6354-445X)

Abstract: This study aimed to assess the genetic variability in *Aegle marmelos* Correa to develop trait-specific genotypes based on morphological and qualitative traits. The evaluation focused on both morphological and qualitative characteristics within the gene pool of this species. High phenotypic coefficient of variation (PCV) and genotypic coefficient of variation (GCV) were observed for traits such as shell weight, fruit weight, and pulp weight, indicating substantial genetic diversity and strong potential for selective breeding within the germplasm. Heritability estimates ranged widely, with fruit weight showing a low 0.07% and shell weight a

high 92.23%, reflecting the significant impact of environmental factors on trait expression. Principal Component Analysis (PCA) revealed that the first principal component (PC1) explained 40.19% of the total variation, with an eigenvalue of 8.12. The first six principal components collectively accounted for 80.77% of total variability. Genotypes CHESB-25 and CHESB-29 exhibited the highest positive PC scores for PC1 and PC2, identifying them as superior selections. Cluster analysis identified six distinct clusters of genotypes, with Cluster V being the largest and Cluster VI the smallest. This clustering highlights the genetic diversity among the bael genotypes and provides a basis for breeding and selection strategies. Cluster IV emerged as the most promising, consistently showing the highest values for key attributes such as shell weight, fruit weight, and fruit yield per plant. Therefore, prioritizing Cluster IV is recommended for selecting superior varieties and developing new cultivars. The study also noted that fruit yield per plant positively correlated with traits like shell weight and fruit weight, emphasizing the importance of these traits for yield improvement. Conversely, negative correlations with seed percent, shell percent, and phenolic content suggest these traits may be less beneficial for enhancing yield. The hierarchical clustering heat map of the 101 bael germplasms offers a detailed perspective on the relationships between various traits and germplasms. The results offer vital information for creating A. marmelos cultivars with higher yields and better quality. For breeding programs, targeted selection is made possible by the discovery of important clusters and superior genotypes (CHESB-25 and CHESB-29). Given the high level of genetic variation found, hybridization may be able to improve desired characteristics like fruit output and weight. Overall, the findings offer important insights for selecting elite genotypes and advancing breeding programs.

Keywords: *Aegle marmelos*, Genetic diversity, Heritability, GCV, PCA, Cluster analysis

Introduction

The bael tree (*Aegle marmelos* Correa), an ancient species from India, belongs to the Rutaceae family. Though often underappreciated, it has been known since prehistoric times and holds a revered place in Hindu mythology. Hindus consider this tree sacred, offering its fruits and leaves to deities such as Goddess Parvati and Lord Shiva. Its medicinal value is well-documented in ancient Indian texts, including *Upvana Vinod*, the Sanskrit *Charaka Samhita*, and *Brihat Samhita*. The fruit is also mentioned in the Yajur Veda (Om Prakash, 1961). Bael's rich history of therapeutic use is further illustrated by its depiction in *Ajanta* cave paintings and references in Buddhist and Jain literature (Singh *et al.*,2024[a]). Keeping the above facts in the background, a large number of germplasms were collected and established in the field gene bank of the station clonally.

Bael thrives in areas with poor water sources and offers a wealth of functional vitamins and nutrients. As the global demand for superfoods and natural antioxidants rises, bael presents a significant opportunity for developing innovative food products (Singh *et al*.,2020[b]). It is used to create a range of value-added items, including squash, murabba, fruit slabs, toffees, powders, and jams (Singh *et al.*,2018[a]; Singh *et al*.,2019[c]). The ripe pulp of premium bael cultivars, as well as the “sherbat” made from it, is highly valued for its mild laxative, tonic, digestive, and restorative properties, and is effective in treating biliousness. Conversely, green, unripe bael fruits are commonly used to prepare “murabba,” a preserve renowned for its digestive benefits. In India, a popular drink called “sherbat” is made by blending seeded bael pulp with milk or water and sugar (Bhattacherjee *et al.*,2015; Singh *et al.*,2018[b]). Although bael fruits are rarely consumed as a dessert due to their astringent taste, numerous seeds, and high fiber content, the pulp contains

marmelosin, which imparts restorative, astringent, and laxative benefits (Pathirana *et al*., 2020). All parts of the bael tree—stem, bark, root, leaf, flower, seed oil, and fruit at any stage of maturity—are used in Ayurvedic medicine. The fruit and leaves are known for their proven antioxidant, cardiotonic, antifungal, and analgesic properties. Bael is naturally found in the sub-Himalayan region of India, as well as in dry deciduous forests in central and southern India (Pandey *et al*., 2008). Various landraces of bael are distributed across a wide range of environments, including tropical, arid, semi-arid, and dry subtropical zones. Bael genotypes are notably prevalent in the plains of Gujarat, Rajasthan, and Uttar Pradesh, thriving particularly in arid climates, hilly areas, woodlands, and wastelands (Singh *et al.,*2023; Singh *et al.,*2011) To identify superior variants, it is essential to evaluate a diverse range of genotypes under rainfed semi-arid conditions, as significant variation in both qualitative and quantitative traits is observed among them (Singh *et al.,*2015*;* Singh *et al.,*2020[a]).

In traditional Indian medicine, the root, bark, leaves, flowers, and fruit of the bael tree are used for their ethnopharmacological properties, including anti-diabetic, antiviral, antifungal, and anticancer effects (Singh *et al.,*2019[b]; Singh *et al.,*2014[b]). Bioactive compounds isolated from these parts show potential for treating various human diseases and conditions (Singh 2019; Singh *et al.,*2021[a]). Bael leaf poultices are applied to ulcers and eye infections, while leaf powder serves as a repellent against storage pests. The bark and mature fruit are utilized in the tanning and dyeing industries, and the leaves are employed in treating beriberi and dropsy (Singh *et al.,*2022; Singh *et al.,*2019[a]). Diluted leaf extracts provide relief from tartar, and research has explored the potential of leaf alkaloids in treating asthma (Singh *et al.,*2014[a]; Singh *et al.,*2000). The young leaves and tender shoots are used as animal fodder, and the gum from the stem and

seed locules is used to stabilize drilling fluids. Additionally, bael wood is used for making small agricultural tools and as pulp for wrapping paper (Pandey *et al.*,2020; Singh *et al.*,2021[b];).

The use of morpho-pomological and biochemical profiling allows for a comprehensive evaluation of genetic diversity and agronomic traits in bael, which is critical for the conservation of its germplasm and the planning of an effective breeding program (Singh et al., 2019a).

In this investigation, we analyzed the morphometric, growth, yield, and quality diversity within the bael gene pool to identify elite genotypes specifically adapted to rainfed semi-arid conditions in western India. Our efforts focused on characterizing various traits to pinpoint superior genotypes suited for these challenging environmental conditions. The study aimed to uncover genetic variations that could enhance bael cultivation under rainfed conditions. By evaluating these traits, we sought to identify and promote the best-performing bael genotypes for improved crop performance in semi-arid regions. To enhance variety quality in all horticultural aspects, the presence of genetic variability within a population is essential for the planning and execution of effective crop improvement programs (Singh et al., 2020). Greater variability in crop plants offers an opportunity to identify the genotypes having desirable traits to fulfill the needs of the stakeholders (Singh et al., 2021a). Understanding the genetic diversity within bael germplasm is key to advancing crop improvement efforts and developing targeted breeding programs.

Material and methods

The present study was carried out at the Bael Germplasm Experimental Block of the National Active Germplasm Site, situated at the Central Horticultural Experiment Station (ICAR-CIAH) in Godhra, India 2022-2024. The location is at latitude 22°41'38" N, longitude 73°33'22" E, and an elevation of 113 meters above sea level. This station maintains the world's largest

germplasms of bael (217 clonal and 121 seedlings). The collection sites of bael genotypes have been presented in Fig. 1. Budded bael trees were raised on seedling rootstocks developed from seeds of a single plant, spaced at 7 m ×6 m. The age of the trees in the orchard is 12 years. Each genotype was represented by six plants, with two plants serving as a replication. The planting layout adhered to a randomized block design. The experimental site was characterized by loam to clay loam having151.25, 8.22 and 143.50 kg/ha available nitrogen, phosphorus, and potassium, respectively, at 0-15 cm soil depth with pH 7.50, EC 1.14dS/m, bulk density1.42g/cc, hydraulic conductivity0.28cm/h and organic carbon 0.33%. During the experiment, a uniform dose of 50 kg of farmyard manure /tree was applied and cultivated purely under rainfed conditions in Gujarat. The experimental site is characterized by a semi-arid, hot climate with an average annual precipitation of 750 mm and significant temperature fluctuations, ranging from 35-46°C in summer to 9-25°C in winter (Singh et al., 2024; Yadav et al., 2024). The standard package of practices was followed according to Singh et al.(2019a).

The samples of marketable fruits were harvested from pest and disease-free healthy plants for different observations and analyses. The samples were washed with tap water and excess water was drained. Fresh samples of fruit were used for the determination of Total soluble solids (TSS) and acidity. TSS of fruits and mucilage were determined using a digital refractometer and results were expressed as °Brix (Singh et al. 2016c; Yadav et al., 2022). The pulp acidity contents of the samples were determined by visual titration with 0.1 N sodium hydroxide (NaOH) to an endpoint of pH 8.1 as suggested by Ranganna (1986).

Total phenolics were estimated using the Folin–Ciocalteu reagent (Singleton et al. 1999). Results were expressed as gallic acid equivalent (mg GAE/100 g fresh weight, FW). Ascorbic acid

content was determined in accordance with the dinitrophenylhydrazine (DNPH) method. The value was expressed as mg/100 g FW (Yadav et al., 2022; Yadav et al., 2024).

Data analysis

Burton's (1952) method is followed in the computation of the variance components and coefficients of variation. One derived expected general genetic progress and heritability using the formulae Johnson et al. (1955). Al-Jibouri et al. (1958) developed a technique for evaluating all possible feature combinations by assessing correlation coefficients at both phenotypic and genotypic levels, focusing on variances in these coefficients. RStudio (version 2023.3.0+) tools were used for Data processing and graphical visualization. Principal component analysis (PCA) and facetted density plots were performed using the most current available versions of the FactoMineR, factoextra, and ggplot2 packages, drawing on fundamental methods by Hotelling (1933) and Pearson (1901). Cluster analysis was conducted with the cluster, factoextra, dendextend, and ggplot2 programs following Sokal and Michener (1958). Variance analysis used the variability and agriculture packages, while correlation analysis was conducted with the corrplot package (Posit team, 2023). Additionally, ordinary least squares (OLS) regression analysis was employed to investigate the relationship between the selected characteristics and fruit yield per plant. The results from this regression analysis highlight the significance of these characteristics in predicting yield outcomes, as they offer a model that explains a substantial portion of the variance in fruit yield per plant.

Results and discussion

Morphological variations among *genotypes*

The evaluation of the *Aegle marmelos* gene pool under rainfed semi-arid conditions is significant for several reasons. It provides empirical evidence on how different genotypes perform regarding growth, yield, and quality traits in challenging environments. This research is crucial for identifying suitable varieties and elite genotypes that can thrive in arid and semi-arid conditions, which is essential for effective crop improvement and adaptation strategies. Observations of prominent morphometric variability in the *A. marmelos* germplasm—such as differences in growth patterns, fruit characteristics, and quality attributes—are illustrated through various Figs. These include variability in fruit and growth traits (Figs. 2-4), genetic diversity in fruit components (Figs. 5-6), and variations in locule arrangement, seed characteristics, and fiber color (Figs. 7-9). Additionally, variations in fruit size and shape, thorn orientation, stigma color, and stem bark color (Figs. 10-13) highlight the diverse potential within the gene pool. This detailed documentation underscores the importance of understanding these variations for selecting and breeding elite genotypes suited for semi-arid conditions.

Genetic variability studies

Estimates of genetic parameters for various yield traits in bael are presented in Table 1. High phenotypic coefficient of variation (PCV) and genotypic coefficient of variation (GCV) were observed for shell weight (27.29 and 27.21), fruit weight (30.27 and 30.14), pulp weight (33.83 and 33.79), shell thickness (20.16 and 20.12), number of seeds per fruit (29.26 and 29.24), fiber weight (29.00 and 28.21), seed percentage (30.89 and 30.74), shell percentage (22.71 and 22.68), and phenolic content (20.99 and 20.82). These high PCV and GCV values indicate substantial variability within the germplasm, suggesting strong potential for selection (Bhaskara *et al.*,2011; Singh *et al.*,2024[b]; Di Matteo *et al.*,2021). Moderate PCV and GCV were found for fruit

circumference (16.99 and 16.83), total seed weight (17.05 and 17.01), vitamin C (17.44 and 17.41), non-reducing sugar (10.08 and 10.01), fruit length (16.24 and 16.21), fruit width (16.99 and 16.86), and fruit yield per plant (13.13 and 13.11), indicating a moderate level of genetic variability in these traits. In contrast, low PCV and GCV values were recorded for pulp percentage (5.69 and 5.57) and TSS pulp (7.92 and 7.87), reflecting minimal variability within the evaluated germplasm for these traits (Kumar *et al.*,2023; Norouzi *et al.*,2017; Singh *et al.*,2010).

High broad-sense heritability supports the identification of favorable traits for selection, allowing breeders to focus on superior genotypes based on their phenotypic expression of quantitative traits. Heritability ranged from 0.07% for fruit weight to 92.23% for shell weight, indicating that environmental factors largely influence these traits. Genetic advances as a percentage of the mean ranged from 0.39% for total sugar to 25.07% for fiber weight. Notably, high genetic advances were observed for fiber weight (25.07%), while moderate advances were noted for shell weight (11.92%) and number of seeds per fruit (17.99%). High heritability coupled with low genetic advances as a percentage of the mean for shell weight suggests non-additive gene action and limited selection response. Conversely, low heritability and genetic advances for fruit weight (0.07% and 1.36%), pulp weight (0.06% and 1.68%), pulp percentage (7.05% and 0.50%), shell thickness (0.10% and 0.56%), fruit circumference (14.98% and 6.27%), fruit width (1.75% and 2.32%), fruit length (2.17% and 2.32%), total seed weight (7.16% and 4.51%), seed percentage (0.14% and 2.00%), shell percentage (3.98% and 5.98%), TSS pulp (2.99% and 0.61%), vitamin C (3.74% and 3.47%), acidity (10.04% and 6.83%), total sugar (0.95% and 0.39%), phenolic content (11.07% and 8.32%), and non-reducing sugar (1.09% and 0.64%) indicate that these traits are highly influenced by environmental factors and are less responsive to

selection (Amulya *et al.*,2022; Ganopoulos *et al.*,2016; Singh *et al.*,2010, Khadivi et al., 2024, Gürcan et al., 2024).

Character association studies

The genotypic correlation coefficients among various characters of bael genotypes, as detailed in Table 2 and Fig. 14 reveal several key insights. Fruit yield per plant demonstrated positive correlations with shell weight (0.55), fruit weight (0.78), pulp weight (0.77), pulp percentage (0.65), shell thickness (0.31), fruit circumference (0.70), fruit width (0.70), fruit length (0.67), number of seeds per fruit (0.46), total seed weight (0.39), fiber weight (0.11), TSS pulp (0.01), vitamin C (0.09), acidity (0.06), and total sugar (0.01). Thus, these traits should be prioritized to enhance yield per plant. Conversely, negative correlations were observed with seed percentage (-0.73), shell percentage (-0.52), phenolic content (-0.08), and non-reducing sugar (-0.01) (Singh *et al.*, 2024[a]; Uddin *et al.*, 2016). Non-reducing sugar positively correlated with pulp percentage (0.04), shell thickness (0.10), number of seeds per fruit (0.02), fiber weight (0.15), TSS pulp (0.01), total sugar (0.91), and phenolic content (0.20), but negatively with shell weight (-0.05), fruit weight (-0.01), pulp weight (-0.01), fruit circumference (-0.09), fruit width (-0.09), fruit length (-0.004), total seed weight (-0.04), shell percentage (-0.04), seed percentage (-0.06), vitamin C (-0.21), and acidity (-0.004). Phenolic content was positively associated with pulp percentage (0.04), shell thickness (0.03), vitamin C (0.08), acidity (0.05), and total sugar (0.22), while negatively correlated with shell weight (-0.12), fruit weight (-0.10), pulp weight (-0.09), fruit circumference (-0.24), fruit width (-0.24), fruit length (-0.15), number of seeds per fruit (-0.16), total seed weight (-0.27), fiber weight (-0.07), shell percentage (-0.04), seed percentage (-0.08), and TSS pulp (-0.06). Total sugar showed positive correlations with pulp percentage

(0.003), shell thickness (0.03), fruit length (0.002), fiber weight (0.05), TSS pulp (0.04), and acidity (0.01), but negative correlations with shell weight (-0.05), fruit weight (-0.04), pulp weight (-0.03), fruit circumference (-0.06), fruit width (-0.06), number of seeds per fruit (-0.09), total seed weight (-0.17), shell percentage (-0.01), seed percentage (-0.10), and vitamin C (-0.15) (Krishna *et al.*,2022; Peixoto *et al.*,2021; Singh *et al.*,2024[a])

Acidity was positively correlated with fruit weight (0.01), pulp weight (0.01), fruit circumference (0.01), fruit width (0.01), fruit length (0.03), number of seeds per fruit (0.06), total seed weight (0.05), TSS pulp (0.08), and vitamin C (0.14), but negatively with shell weight (-0.02), shell thickness (-0.03), fiber weight (-0.03), seed percentage (-0.003), and shell percentage (-0.04). Vitamin C exhibited positive associations with fruit weight (0.01), pulp weight (0.03), pulp percentage (0.21), fruit length (0.004), and TSS pulp (0.16), while negatively correlated with shell weight (-0.13), shell thickness (-0.14), fruit circumference (-0.01), fruit width (-0.01), number of seeds per fruit (-0.05), total seed weight (-0.01), fiber weight (-0.15), seed percentage (-0.02), and shell percentage (-0.20). TSS pulp showed positive correlations with shell weight (0.08), fruit weight (0.04), pulp weight (0.04), shell thickness (0.01), fruit circumference (0.18), fruit width (0.18), fruit length (0.12), number of seeds per fruit (0.14), total seed weight (0.22), seed percentage (0.10), and shell percentage (0.06), but negative correlations with pulp percentage (-0.01) and fiber weight (-0.14). Shell percentage was positively correlated with shell weight (0.17), shell thickness (0.02), and seed percentage (0.56), but negatively with fruit weight (-0.53), pulp weight (-0.61), pulp percentage (-0.95), fruit circumference (-0.41), fruit width (-0.41), fruit length (-0.42), number of seeds per fruit (-0.27), total seed weight (-0.24), and fiber weight (-0.02). Seed percentage positively correlated only with total seed weight (0.03), but negatively with shell weight (-0.48), fruit weight (-0.71), pulp weight (-0.72), pulp percentage (-

0.70), shell thickness (-0.28), fruit circumference (-0.58), fruit width (-0.58), fruit length (-0.61), number of seeds per fruit (-0.12), and fiber weight (-0.09). Fiber weight showed positive correlations with shell weight (0.08), fruit weight (0.06), pulp weight (0.03), shell thickness (0.16), fruit length (0.01), number of seeds per fruit (0.06), and total seed weight (0.10), but negative correlations with pulp percentage (-0.15), fruit circumference (-0.02), and fruit width (-0.02). Total seed weight was positively associated with shell weight (0.45), fruit weight (0.60), pulp weight (0.58), pulp percentage (0.28), shell thickness (0.34), fruit circumference (0.44), fruit width (0.60), fruit length (0.60), and number of seeds per fruit (0.59). The number of seeds per fruit correlated positively with shell weight (0.50), fruit weight (0.67), pulp weight (0.66), pulp percentage (0.34), shell thickness (0.44), fruit circumference (0.60), fruit width (0.60), and fruit length (0.59). Fruit length and fruit width exhibited positive associations with shell weight (0.64 and 0.65), fruit weight (0.85 and 0.86), pulp weight (0.84 and 0.85), pulp percentage (0.57 and 0.57) and shell thickness (0.33 and 0.33). Finally, fruit circumference showed positive correlations with shell weight (0.65), fruit weight (0.86), pulp weight (0.85), pulp percentage (0.57) and shell thickness (0.33), while shell thickness positively correlated with shell weight (0.59), fruit weight (0.53), pulp weight (0.49) and pulp percentage (0.07). The pulp percent showed positive associations with shell weight (0.07), fruit weight (0.68), and pulp weight (0.74) at genotypic levels. Pulp weight exhibited positive correlations with both shell weight (0.64) and fruit weight (0.99) at genotypic levels. The fruit weight showed a positive association with shell weight (0.72) at genotypic levels (Verma *et al.*,2024; Singh *et al.*,2024[a], Khadivi et al., 2025, Gürcan et al., 2024).

Overall, the most significant traits for improving fruit yield per plant are shell weight, fruit weight, pulp weight, pulp percentage, shell thickness, fruit circumference, fruit width, and fruit

length. Additionally, quality parameters like TSS pulp, vitamin C, acidity, and total sugar are also important for enhancing fruit quality (Schnell *et al.*,2001; de Jesus *et al.*,2022; Singh *et al.*,2024[a], Khadivi et al., 2025,).

Principal component analysis

The eigenvalues and their percentage variations are detailed in Table 3 and Fig. 15. As the number of principal components (PCs) increases, the eigenvalue and variance associated with each principal component decrease, while the cumulative variability increases. The scree plot illustrates the percentage of variation explained by each PC, showing that the first principal component accounts for approximately 40.19% of the variability with the highest eigenvalue of 8.12 (Fig. 15). Subsequent principal components exhibit a progressive decrease in eigenvalue: the second PC has an eigenvalue of 2.32, the third PC 2.14, the fourth PC 1.47, the fifth PC 1.19, and the sixth PC 1.08. The percentage of total variation explained by these PCs also decreases, with PC-2 explaining 11.49%, PC-3 10.60%, PC-4 7.25%, PC-5 5.88%, and PC-6 5.36%. Among the twelve PCs identified, only the first six (PC1 to PC6) have eigenvalues greater than 1.00, indicating that these components capture significant traits influencing the phenotype of the individuals. Together, they account for approximately 80.77% of the total variation observed across the 101 cultivars for the 20 traits studied (Debbarma *et al.*,2024; Dhakar *et al.*,2019; Singh *et al.*,2024[a]; Khadivi et al., 2024; Gürcan et al., 2024).

The PC scores for 101 bael genotypes

In principal component analysis (PCA), PC scores represent the projection of the original data onto the principal components. These scores quantify each variable's contribution to the principal

components and help identify which variables are most influential in explaining the data's variation. Additionally, PC scores are instrumental in various analyses, such as clustering, classification, and regression, enabling the exploration of relationships between variables and principal components (Singh *et al.*,2024[a]; Debbarma *et al.*,2024, Khadivi et al., 2025, Sümbül et al., 2025). The PC scores for both the first principal component (PC1) and the second principal component (PC2) include both positive and negative values (Table 4).

For the first principal component, the highest positive PC score was recorded for CHESB-25 (146.64), followed by CHESB-29(129.25), CHESB-39(99.23), and CHESB-45 (99.05). The lowest positive score in this component was (4.74) for CHESB-13. Conversely, the negative PC scores ranged from -0.04 for CHESB-74 to -134.50 for CHESB-12. In the second principal component, the highest positive PC score was (156.65) for CHESB-25, while the lowest positive score was (0.35) for CHESB-97. The negative PC scores varied from (-0.03) for CHESB-61 to (-97.67) for CHESB-60.

The relationship between each trait and PC1 *vs.* PC2 was presented in Fig. 16. In first and second principal component, CHESB-4, CHESB-13, CHESB-17, CHESB-18, CHESB-19, CHESB-20, CHESB-23, CHESB-25, CHESB-31, CHESB-37, CHESB-43, CHESB-53, CHESB-56, CHESB-58, CHESB-62, CHESB-68, CHESB-70, CHESB-72, CHESB-76, CHESB-77, CHESB-81, CHESB-83, CHESB-88, CHESB-89, CHESB-91, CHESB-93 and CHESB-96 depicted positive active genotypes. The contribution of these genotypes was greater for the variability in both PC1 and PC2.

Hierarchical clustering analysis

Hierarchical cluster analysis (HCA) is essential for identifying superior genotypes and tailoring breeding strategies. The analysis resulted in six clusters comprising 101 genotypes Table 5 and Fig. 17. Cluster V was the largest, including 36 genotypes(CHESB-1, CHESB-4, CHESB-5, CHESB-6,CHESB-8, CHESB-11, CHESB-13, CHESB-15, CHESB-16, CHESB-17, CHESB-19, CHESB-20, CHESB-21 , CHESB-25, CHESB-27, CHESB-37, CHESB-43, CHESB-46, CHESB-49, CHESB-53, CHESB-56, CHESB-58, CHESB-62, CHESB-63, CHESB-65, CHESB-66, CHESB-68, CHESB-70, CHESB-72, CHESB-74, CHESB-76, CHESB-77, CHESB-78, CHESB-88, CHESB-91, CHESB-96), followed by Cluster II with 24 genotypes(CHESB-7, CHESB-22, CHESB-24, CHESB-30, CHESB-32, CHESB-35, CHESB-38, CHESB-40, CHESB-44, CHESB-47, CHESB-54, CHESB-55, CHESB-57, CHESB-60, CHESB-80, CHESB-82, CHESB-84, CHESB-86, CHESB-89, CHESB-92, CHESB-94, CHESB-97, CHESB-99, CHESB-101), Cluster III with 19 genotypes(CHESB-3, CHESB-18, CHESB-23, CHESB-29, CHESB-34, CHESB-39, CHESB-41, CHESB-45, CHESB-50, CHESB-52, CHESB-71, CHESB-73, CHESB-79, CHESB-81, CHESB-83, CHESB-85, CHESB-93, CHESB-95, CHESB-98), and Cluster I with 12 genotypes(CHESB-2, CHESB-9, CHESB-10, CHESB-14, CHESB-26, CHESB-31, CHESB-33, CHESB-42, CHESB-48, CHESB-59, CHESB-64, CHESB-69). Cluster IV contained 9 genotypes (CHESB-12, CHESB-28, CHESB-36, CHESB-61, CHESB-67, CHESB-75, CHESB-87, CHESB-90, CHESB-100), while Cluster VI had only a single genotype (CHESB-51). This clustering provides valuable insights into the genetic diversity among the studied cultivars, revealing distinct groupings based on phenotypic traits and offering important implications for breeding and selection strategies (Fig.17). (Ahmed *et al*.,2011; Saran *et al.*,2022; Singh *et al.*,2024a*;*) The relationships among the different genotypes are illustrated in the dendrogram. Genotypes clustered closely together in the dendrogram are more like one another

compared with those farther apart. Additionally, the dendrogram displays the relative similarity among genotypes across the various clusters (Singh *et al.*,2024a; Debbarma *et al.*,2024; Dhakar *et al.*,2019, Yaman et al., 2024, Khadivi et al., 2024, Gürcan et al., 2024).

Hierarchical Clustering Heatmap (HCH) represents twenty-yield and quality characters of the 101bael genotype (Fig. 18). Based on the Hierarchical Clustering Heatmap (HCH), five groups were created by loosely organizing the one-hundred-one *A. marmelos* germplasm. Among the five groups, group V had the maximum number of genotypes (71), followed by group IV (18). The lowest genotype was found in group II (1), followed by group I (2). The hierarchical clustering heat map shows the linkages and trends among numerous dataset attributes and samples. The heatmap displaying the standardized characteristics values uses color gradients; darker colors imply lower values and brighter hues suggest higher values. Whereas the left dendrogram unites similar traffic samples, the top dendrogram clusters similar traits based on their expression patterns across samples. This visualization enables one to identify groupings of characteristics with regular fluctuations and groups of samples with similar qualities. Such patterns would enable one to grasp the primary organization of the data by revealing connections and similarities that might direct environmental investigations, genetic research, and breeding plans. Analyzing the clusters and color patterns allows researchers to understand the relationships between properties and identify significant characteristics separating different samples. The acidity% in CHESB-50 is a primary trait separating high-performing germplasms from others and may be focused on further breeding and genetic studies of bael. Yield-related traits like fruit weight, pulp weight, and number of seeds per fruit directly influence the overall yield per plant, indicating better yield potential. Likewise, some quality traits, including pulp Percentage, TSS, vitamin C, and acidity, significantly impact the overall quality of bael, and

physical characteristics, including shell weight and thickness, provided insights into the overall fruit structure and marketability. Studying the genetic relationships between germplasm is made more accessible by cluster analysis, which may put germplasm with comparable genetic information into one group (Singh et al., 2024; Yadav et al., 2024). Saran et al., 2022 also mentioned that the two-dimensional plot generated from PCA showed three groups that were similar to the clustering pattern of the UPGMA dendrogram.

Cluster mean analysis

Clusters mean analysis exhibits ample variation for all the growth and yield attributing characters and were presented (Table 6). The shell weight of the fruit exhibited the highest cluster mean value in Cluster IV (304.51), followed by Cluster II (256.37), with the lowest value recorded in Cluster III (149.42). For fruit weight, the maximum cluster mean of 2.48 was observed in Cluster IV, followed by Cluster II at 1.62, while the minimum mean was noted in Cluster III at 0.84. The highest cluster mean for pulp weight was found in Cluster IV (2.10), with Cluster II following at 1.29, and the lowest mean observed in Cluster III (0.63). Regarding pulp percentage, Cluster IV had the highest mean value at 84.69%, with Cluster V close behind at 83.12%, while Cluster III had the lowest mean value at 74.46%. Shell thickness had the highest cluster mean values in Cluster IV (3.37) and Cluster II (3.03), with the lowest mean recorded in Cluster I (2.29). The highest cluster mean for fruit circumference was found in Cluster IV at 54.18, followed by Cluster I at 43.36, while Cluster III had the lowest mean at 30.49. For fruit width, the maximum cluster mean of 17.24 was observed in Cluster IV, with Cluster I at 13.80 and the minimum mean in Cluster III at 9.70. Fruit length had the highest cluster mean in Cluster IV at 19.51, followed by Cluster VI at 15.84, with the lowest mean observed in Cluster III at 11.72. The number of

seeds per fruit showed the highest cluster mean in Cluster IV at 211.74, followed by Cluster VI at 165.98, while Cluster III recorded the lowest mean at 96.65 (Debbarma *et al.*,2024; Singh *et al.*,2024a).

The total seed weight was highest in Cluster IV (34.14) and Cluster I (29.44), with the lowest mean observed in Cluster III (22.18). The highest cluster means for fiber weight was noted in Cluster I (46.82) and Cluster II (44.94), whereas Cluster VI recorded the lowest value at 38.34. Cluster III had the highest mean for seed percentage at 2.72, followed by Cluster I at 2.31, while Cluster IV had the lowest mean at 1.38. The shell percentage was highest in Cluster III (18.06) and Cluster I (17.26), with the lowest mean observed in Cluster V (12.18). The highest cluster means for TSS pulp were found in Cluster VI (40.55) and Cluster I (40.20), while Cluster III had the lowest mean at 37.65. Vitamin C levels were highest in Cluster VI (23.35), followed by Cluster V (19.75), with the lowest value in Cluster II (17.46). The highest cluster mean for acidity was recorded in Cluster II (0.58) and Cluster III (0.57), with the lowest mean in Cluster IV and Cluster V (0.54). For total sugar, the maximum cluster mean was observed in Cluster II (20.93), followed by Cluster III (20.67), while the minimum was recorded in Cluster V (20.08). Phenolic content was highest in Cluster VI (30.62) and Cluster III (29.95), with the lowest mean in Cluster I (20.25). Non-reducing sugar was highest in Cluster II (17.77), followed by Cluster III (17.53), and the lowest in Cluster V (16.68). Finally, the highest cluster mean for fruit yield per plant was observed in Cluster IV (108.99) and Cluster VI (104.56), with the lowest mean in Cluster III (75.45) (Debbarma *et al.*,2024; Singh *et al.*,2024a). Genotypes with the highest average values demonstrate significant potential for selection. Consequently, it is essential to prioritize Cluster IV for selecting inbred varieties and developing new cultivars in future breeding programs.

Regression analysis

The OLS regression results indicate a model that explains a significant proportion of the variance in fruit yield per plant (Table 7). However, many individual predictors do not show statistically significant effects on the yield. Potential issues with multicollinearity might be present, affecting the coefficient estimates' reliability. The model seems overall robust but may benefit from further investigation into multicollinearity and possibly refining the predictor set. This table summarizes the relationship between several predictor variables and the dependent variable, Fruit yield/plant (kg). The model explains approximately 74.9% of the variability in fruit yield per plant. This relatively high value suggests that the model explains a significant portion of the variance. The F-statistic tests present the overall significance of the model, where a high value indicates that the model significantly improves prediction compared with a model with no predictors.

Similarly, a very low p-value of the Prob F-statistic test indicates that the overall model is statistically significant. A positive coefficient (pulp%, fruit width, number of seeds/fruits, total seed weight, fiber weight, shell%, vitamin C, acidity%, and total sugars) indicates a positive relationship with the dependent variable, while a negative coefficient indicates a negative relationship. The total sugar content has a positive association with fruit yield. The lack of statistical significance across most traits suggests that further research is needed to identify the key factors influencing yield in bael genotypes. The overall model fit is moderate, with an R-squared value of 0.749, indicating that while the model can explain some variance in yield, many traits may not significantly impact fruit yield. Fig. 19 represents the association between fiber weight (g) and "Fruit yield/plant (kg) based on the OLS regression model. The red line shows the

regression line, whilst the blue crosses illustrate the simulated data points. This image shows the correlation between fiber weight (g) and Fruit yield/plant (kg) using the regression model coefficients.

Conclusions

The present study highlights the significance of the regional and national bael gene pool in semi-arid conditions by examining variability in morphometric, yield, and quality traits, with a particular emphasis on yield and qualitative attributes. Notably, high phenotypic coefficient of variation (PCV) and genotypic coefficients of variation (GCV) were observed for shell weight, fruit weight, and pulp weight, demonstrating substantial genetic diversity within the germplasm and a strong potential for selective breeding. The heritability of traits varied significantly, from a low of 0.07% for fruit weight to a high of 92.23% for shell weight, indicating varying influences of environmental factors. Principal Component Analysis (PCA) revealed that the first principal component explained about 40.19% of the total variation, with a high eigenvalue of 8.12. The first six principal components collectively explained about 80.77% of the total variability, highlighting their significance in capturing the traits influencing bael phenotypes. The highest positive PC scores for the first and second principal components were observed in genotypes CHESB-25 and CHESB-29, indicating their superior performance in these components. Additionally, based on their performance, CHESB-1, CHESB-8, CHESB-19, CHESB-27, CHESB-34, CHESB-39, CHESB-41, CHESB-45, CHESB-50, CHESB-52, CHESB-53, CHESB-56, CHESB-64, CHESB-71, CHESB-73, CHESB-81, CHESB-83, CHESB-85, CHESB-93, CHESB-95, and CHESB-98 also recorded good scores based on their performance, which could be useful for planning and carrying out breeding programs. Cluster analysis identified six distinct

clusters, with Cluster V being the largest and Cluster VI the smallest. This clustering underscores the genetic diversity among bael genotypes, which can inform breeding and selection strategies. The positive correlations of fruit yield per plant with various traits such as shell weight, fruit weight, and pulp weight emphasize the importance of focusing on these traits to enhance yield. Cluster analysis reveals substantial variation in growth and yield traits across bael genotypes, with Cluster IV consistently showing the highest values for key attributes like shell weight, fruit weight, and yield per plant. Prioritizing Cluster IV is recommended for selecting superior inbred varieties and developing new cultivars in breeding programs. This research provides valuable insights into the extensive natural variation among 101 bael genotypes, emphasizing the significance of identifying elite genotypes within a large gene pool. Such insights are crucial for advancing genetic improvement efforts on both national and global scales.

Ethics approval and consent to participate: Not Applicable

Research involving Human Participants and, or Animals: Not applicable

Clinical Trial Study: Not applicable

Informed consent: Not applicable

Statement on experimental research and field studies on plants

The either cultivated or wild-growing plants sampled comply with relevant institutional, national, and international guidelines and domestic legislation of India.

Consent for publication: Not applicable

Availability of data and materials:

The data that support the findings of this study are available from the corresponding author upon reasonable request.

Competing interests:

The authors declare no conflict of interest.

Funding: Not applicable

Author Contributions: AKS: Conceptualization, methodology, visualization, writing—review and editing. VY, LPY: Conceptualization, writing—original draft preparation, data curation, visualization. KG, AP: Statistical analysis, formal analysis, data curation, writing—review and editing. JR, PR, AS, PK, YT, AK: methodology, visualization, writing—review and editing. All authors have read and agreed to the published version of the manuscript.

References

Ahmed, M. V. O. M., Bouna, Z. E. O., Lemine, F. M. M., Djeh, T. K. O., Mokhtar, T., Salem, A. O. M., 2011. Use of multivariate analysis to assess phenotypic diversity of date palm (*Phoenix dactylifera* L.) cultivars. *Scientia Horticulturae*. 127(3): 367-371.

Al-Jibouri, H. A., Miller, P. A., & Robinson, H. F. (1958). Genotypic and environmental variances and covariances in an upland cotton cross of interspecific origin. Agronomy Journal, 50(10), 633-636.

Amulya, R. N., Adivappar, N., Shivakumar, B. S., Satish, K. M., 2022. Studies on genetic variability and relationship of bael (*Aegle marmelos* (L) Correa) using morphological and molecular markers. *J. Hortic. Sci*. 17, 88–94. doi: 10.24154/jhs.v17i1.846.

Bhaskara, R. K. V., Sekar, D. K., Kumar, G., Karthik, L., 2011. A review on pharmacological and phytochemical properties of *Aegle marmelos* L. Corr. *Asian J. Plant Sci. Res.* 1(2):8–17.

Bhattacherjee, A. K., Dikshit, A., Pandey, D., Tandon, D. K., 2015. High performance liquid chromatographic determination of marmelosin and psoralen in bael (*Aegle marmelos* (L.) Correa) fruit. Journal of Food Science and Technology, 52(1), 597-600. https://doi.org/10.1007/s13197-013-1015-x.

Burten, L. P., Whyburn, W. M., 1952. Minimax solutions of ordinary differential equations. *In Proc. Am. Math. Soc*. 3,794-803.

Burton, G. W. (1952). Quantitative inheritance in grasses. Proceedings of the 6th International Grassland Congress, 1, 277-283.

de Jesus, O. N., Lima, L. K. S., Soares, T. L., da Silva, L. N., dos Santos, I. S., Sampaio, S. R., de Oliveira, E. J., 2022. Phenotypic diversity and alternative methods for characterization and prediction of pulp yield in passion fruit (*Passiflora* spp.) germplasm. *Scientia Horticulturae*. 292, 110573.

Debbarma, P., Hazarika, T. K., 2024. Genetic diversity of Bael [*Aegle marmelos* (L.) Corr.] accessions from north-east India based on principal component and cluster analysis. *Genetic Resources and Crop Evolution*. 71(1), 253-277.

Dhakar, M. K., Das, B., Nath, V., Sarkar, P. K., Singh, A., 2019. Genotypic diversity for fruit characteristics in bael [*Aegle marmelos* (L.) Corr.] based on principal component analysis. *Genet Resour Crop Evolut*. 66 (4), 951–964.

Di Matteo, A., Simeone, G. D. R., Cirillo, A., Rao, M. A., Di Vaio, C., 2021. Morphological characteristics, ascorbic acid and antioxidant activity during fruit ripening of four lemon (*Citrus limon* (L.) Burm. F.) cultivars. *Scientia Horticulturae*. 276, 109741.

Ganopoulos, I., Moysiadis, T., Xanthopoulou, A., Osathanunkul, M., Madesis, P., Zambounis, A., Kazantzis, K., 2016. Morpho-physiological diversity in the collection of sour cherry

(*Prunus cerasus*) cultivars of the Fruit Genebank in Naoussa, Greece using multivariate analysis. *Scientia Horticulturae.* 207, 225-232.

Gürcan, K., Yılmaz, K. U., Tunç, Y., Yaman, M., Güneş, A., Yıldız, E., ... & Khadivi, A. (2024). Evaluation of genetic diversity in some hybrid individuals of honeyberry (Lonicera caerulea L.) based on fruit characteristics, leaf morphology, vitamin C, antioxidant activity, and biochemical and nutritional contents. BMC Plant Biology, 24(1), 1158. https://doi.org/10.1186/s12870-024-05846-3

Hotelling, H. (1933). Analysis of a complex of statistical variables into principal components. Journal of Educational Psychology, 24(6), 417-441.
Pearson, K. (1901). On lines and planes of closest fit to systems of points in space. Philosophical Magazine, 2(11), 559-572.

Johnson, H. W., Robinson, H. F., & Comstock, R. E. (1955). Estimates of genetic and environmental variability in soybeans. Agronomy Journal, 47(7), 314-318.

Johnson, H. W., Robinson, H. F., Comstock, R. E., 1955. Estimates of genetic and environmental variability in soybeans.

Khadivi, A., Mirheidari, F., Saeidifar, A., Moradi, Y., & Tunç, Y. (2025). Morphological and pomological characterizations of pomegranate (*Punica granatum* L.) to select superior genotypes. Scientific Reports, 15(1), 7038. https://doi.org/10.1038/s41598-025-91595-8

Khadivi, A., Mirheidari, F., Saeidifar, A., Moradi, Y., & Tunç, Y. (2025). Morphological and pomological variations of *Pistacia atlantica* Desf. subsp. *cabulica* and subsp. *mutica* in Sistan-va-Baluchestan province, Iran. BMC Plant Biology, 25(1), 248. https://doi.org/10.1186/s12870-025-06268-5

Khadivi, A., Mirheidari, F., Saeidifar, A., Moradi, Y., & Tunç, Y. (2024). Multivariate analysis of morphological variables in tamarind (Tamarindus indica L.). BMC Plant Biology, 24(1), 1154. https://doi.org/10.1186/s12870-024-05872-1

Krishna, H., Sarolia, D., Parashar, A., Kumar, L., 2022. Descriptors for characterization and evaluation of bael (*Aegle marmelos* Correa) germplasm for evaluation and crop improvement. *Fruits*. 72(2):1–10

Kumar, M., Sharma, S., Kumar, M., Nibhoria, A., Yadav, B. S., 2023. Variability for phenological traits and fruit yield attributes in bael (*Aegle marmelos* Correa) cultivars under semi-arid conditions. *Ekin J Crop Breed Genet* 9(2):119–125.

Norouzi, E., Erfani-Moghadam, J., Fazeli, A., Khadivi, A., 2017. Morphological variability within and among three species of Ziziphus genus using multivariate analysis. *Scientia Horticulturae*. 222, 180-186.

Pandey, D., Misra, A. K., Shukla, P. K., Gundappa, G. N., 2020. Production, protection and processing of bael. Technical bulletin Pub. ICAR-Central Institute for Subtropical Horticulture, Lucknow, 1-40.

Pandey, D., Shukla, S. K., Pandey, G., 2008. Bael (*Aegle marmelos* Correa). Underutilized Horticultural Crops, 3, 201-224.

Pathirana, C. K., Ranaweera, L. T., Madhujith, T., Ketipearachchi, K. W., Gamlath, K. L., Eeswara, J. P., Sooriyapathirana, S. S., 2020. Assessment of the elite accessions of bael [*Aegle marmelos* (L.) Corr.] in Sri Lanka based on morphometric, organoleptic, and elemental properties of the fruits and phylogenetic relationships. *Plos one*, 15(5), e0233609. https://doi.org/10.1371/journal.pone.0233609.

Peixoto, M. A., Malikouski, R. G., Evangelista, J. S. P. C., Alves, R. S., de Morais, A. L., Barbosa, D. H. S. G., Zucoloto, M., Bhering, L. L., 2021. Multitrait and multiharvest analyses for genetic assessment and selection of Tahiti acid lime genotypes through Bayesian inference. *Scientia Horticulturae*. 290, 110536.

Posit Team (2023). RStudio: Integrated Development for R. RStudio, PBC, Boston, MA. Retrieved from http://www.rstudio.com/gr.

Rao, C. R., 1952. Advanced statistical methods in Biometrical Research. John Wiley and sons, New York. p 328.

Saran, P. L., Gajbhiye, N., Patel, R. B., Choudhary, R., 2022. Evaluation of Elite Accessions of *Aegle marmelos* (L.) Correa for Commercial Yield and Quality Parameters Under Indian Conditions. *Erwerbs-Obstbau*. 64(3), 345-356.

Schnell, R. J., Olano, C. T., Campbell, R. J., Brown, J. S., 2001. AFLP analysis of genetic diversity within a jackfruit germplasm collection. *Scientia Horticulturae*, 91(3-4), 261-274.

Singh, A. K., 2019. Bael. In: Handbook of Horticulture Vol. 1, Verma A S, Dutt Som and Pradhan S (Eds.). ICAR-DKMA, New Delhi, pp. 258-261.

Singh, A. K., Mishra, D. S., Sharma, B. D., 2022. Seventy five years of research and development in arid and semi-arid fruit crops. *Int. J. Innov. Hortic*. 11(2), 214-227.

Singh, A. K., Pandey, D., Kumar, R., Yadav, V., Yadav, L. P., Gangadhara, K., Rane J., Kumar, R., Hare, K., 2024. Descriptors for characterization and evaluation of bael (*Aegle marmelos* (L.) Correa ex Roxb.) germplasm for utilization in crop improvement. *Genet Resour Crop Evol*. 1-30 https://doi.org/10.1007/s10722-024-01903-w

Singh, A. K., Pandey, D., Singh, S., Singh, R. S., Misra, A. K., Singh, R. K., 2019[a]. The Bael in India. Published by ICAR-DKMA, New Delhi, pp. 1-130. ISBN: 978-81-7164-186-4.

Singh, A. K., Saroj, P. L., Singh, R. S., 2021[a]. Bael. In: Underutilized Fruits of India (Eds. Singh, R.S., Singh, A.K., Maheshwari, S.K., Bhargava, R., Saroj, P.L.,) Brillion Publishing, New Delhi, pp. 253-270.

Singh, A. K., Singh S., Saroj, P. L., Mishra, D.S., Vikas, Y., Raj, K., 2020[a]. Underutilized fruit crops of hot semi-arid region: Issues and Challenges- a review. *Current Horticulture* 8(1), 12-23.

Singh, A. K., Singh, S., Makwana, P., 2015. Intervarietal morphological variability in bael (*Aegle marmelos*) under rainfed semi-arid hot ecosystem of western India. *Current Horticulture*. 3(2), 3-9.

Singh, A. K., Singh, S., Mishra, D. S., Yadav, V., 2019[b]. Bael. In: Breeding of Horticultural Crops Vol. 2: Tropical Fruits (Eds. Parthasarthy, V.A., Singh S.K., Vinoth, S., Aswath, C.,) Pub. Today and Tommorrow's Publishers, New Delhi, pp. 35-58.

Singh, A. K., Singh, S., More, T. A., 2014[a]. Preliminary evaluation of bael varieties under rainfed conditions of western India. *Indian J. Hort.* 71(2), 264-268.

Singh, A. K., Singh, S., Saroj, P. L., 2018[a]. Bael (Production Technology). Technical Bulletin No.67, Pub. ICAR-CIAH, pp. 1-53.

Singh, A. K., Singh, S., Saroj, P. L., 2018[b]. Exploring morphovariations in bael (*Aegle marmelos*). *Current Horticulture*. 6(2), 52-57.

Singh, A. K., Singh, S., Saroj, P. L., Krishna, H., Singh, R. S., Singh, R. K.,2019[c]. Research status of bael (*Aegle marmelos*) in India: A review. The Indian Journal of Agricultural Sciences, 89(10), 1563-1571. https://doi.org/10.56093/ijas.v89i10.94576.

Singh, A. K., Singh, S., Saroj, P. L., Singh, G. P., 2021[b]. Improvement and production technology of bael (*Aegle marmelose*) in India. *Current Horticulture*. 9 (1) 3-14.

Singh, A. K., Singh, S., Singh, R. S., Joshi, H. K., Sharma, S. K., 2014[b]. Characterization of bael varieties under rainfed hot semi-arid environment of western India. *Indian J. Agric. Sci.*, 84 (10), 1236-42.

Singh, A. K., Singh, S., Singh, R. S., Sharma, B. D., Saroj, P. L., 2023. New bael varieties for dryland. *Indian Horticulture*, *68*(1), 3-6.

Singh, A. K., Singh, S., Singh, R. S., Sisodia, P. S., 2011.The Bael (Potential Underutilized Fruit for Future). Pub. Agro-tech Publishing Agency, Udaipur, Rajasthan, pp. 1-104, ISBN: 978-81-8321-210-6.

Singh, M., Nara, U., Kaur, K., Pandey, V., Pathak, D., Sangha, M.K., Singh, P., 2024[a]. Exploring GGE biplot, stability index, correlation, and clustering analysis for growth and seed yield-related traits in celery (*Apium graveolens* L.). *J. of Applied Research on Med. and Aromatic Plants.* 40, 100546.https://doi.org/10.1016/j.jarmap.2024.100546

Singh, P., Sharma, A., Jasrotia, A., Salgotra, R. K., Sharma, M., Gupta, V., 2024[b]. Diversity in morpho-pomological attributes and biochemical profiling of bael (*Aegle marmelos* (L.) Correa) genotypes of North-Western India. *Heliyon*, 10(4).

Singh, R., Mishra, K.K., Jaiswal, H. R., 2000. Studies on physicochemical characters of fruits of bael genotypes. *Indian J Hortic*. 57 (4), 314–317.

Singh, S., Sharma, J. R., Sehrawat, S. K., Jitarwal, O. P., Gavri, A., 2020[b]. Studies on the collection and evaluation of bael cultivars. *Int. J. Chem. Stud*. 8, 212-214. https://doi.org/10.22271/chemi.2020.v8.i6c.10773.

Singh, V. P., Misra, K. K., 2010. Analysis of genetic variability and heritability in bael (*Aegle marmelos* Correa) germplasm. *Progress Agric*. 10 (1), 132–134.

Sokal, R. R., & Michener, C. D. (1958). A statistical method for evaluating systematic relationships. University of Kansas Science Bulletin, 38, 1409-1438.

Sümbül, A., Uzun, A., Yaman, M., Tunç, Y., Khadivi, A., Keçe, Y. M., ... & Turgunbaev, K. (2025). Antioxidant capacity, biochemical composition, and mineral composition of leaves in two apple species (*Malus domestica* Borkh. and *M. kirghisorum* Al. Fed. & Fed.). *BMC Plant Biology*, *25*(1), 1-17. https://doi.org/10.1186/s12870-025-06284-5

Uddin, M. S., Islam, M. S., Alam, M. A., Hossain, M. M., 2016. Study on physico-morphological characteristics of 14 bael (*Aegle marmelos* Correa.) genotypes grown at Chapainawabganj, Bangladesh. *Int J Minor Fruits Med Aromat Plants.* 2(2):29–33.

Verma, V. K., Rymbai, H., Deshmukh, N. A., Bhattacharjee, B., Jha, A. K., Patel, R. K., Mawlein, J., Deka, B. C Hazarika, S., Mishra, V. K., 2024. Genetic diversity and stability analysis of the improved cultivars of guava and their related species. *Scientia Horticulturae*. 333, 113260.

Yadav, L. P., Gangadhara, K., Apparao, V.V., Singh, A. K.; Rane, J., Kaushik, P., Sekhawat, N., Malhotra, S. K.,Ramniwas, Rai, A. K., Yadav, S. L. and Berwal, M. K. 2024. Nutritional, antioxidants and protein profiling of leaves of *Moringa oleifera* germplasm. South African Journal of Botany 165: 443-454. https://doi.org/10.1016/j.sajb.2024.01.012

Yadav, L. P., Gangdhara, K., Apparao, V.V. 2022. Evaluation of drumstick variety Thar Harsha under rainfed semi-arid conditions for growth, yield and quality along with antioxidant potentiality and nutrient content. South African Journal of Botany, 148, 112-122.https://doi.org/10.1016/j.sajb.2022.04.005.

Yaman, M., Sun, M., Sümbül, A., Demirel, F., Tunç, Y., Khadivi, A., & Yılmaz, K. U. (2024). Multivariate analysis of morphological variation, biochemical content, antioxidant

activity, and molecular characteristics of autochthonous *Berberis crataegina* DC. genotypes in Central Türkiye. BMC Plant Biology, 24(1), 1155. https://doi.org/10.1186/s12870-024-05873-0

Table 1. Estimates of genetic parameters for various characters in bael genotypes

No.	Character	Unit	GCV	PCV	h^2	GA	GAM
1	Shell weight	g	27.21	27.29	92.23	25.17	11.92
2	Fruit weight	kg	30.14	30.27	0.07	0.02	1.36
3	Pulp weight	kg	33.79	33.83	0.06	0.02	1.68
4	Pulp	%	5.57	5.69	7.05	0.40	0.50
5	Shell thickness	mm	20.12	20.16	0.10	0.02	0.56
6	Fruit circumference	cm	16.83	16.99	14.98	2.55	6.27
7	Fruit width	cm	16.86	16.99	1.75	0.30	2.32
8	Fruit length	cm	16.21	16.24	2.17	0.35	2.32
9	No. of seeds/fruit	Number	29.24	29.26	86.19	25.22	17.99
10	Total seed weight	g	17.01	17.05	7.16	1.22	4.51
11	Fiber weight	g	28.21	29.00	36.06	10.46	25.07
12	Seed	%	30.74	30.89	0.14	0.04	2.00
13	Shell	%	22.68	22.71	3.98	0.90	5.98
14	TSS pulp	˚Brix	7.52	7.58	2.99	0.23	0.61
15	Vitamin C	mg/100 g	17.41	17.44	3.74	0.65	3.47
16	Acidity	%	5.17	5.22	10.04	0.05	6.83
17	Total sugar	%	7.87	7.92	0.95	0.08	0.39
18	Phenolic content	mg/g	20.82	20.99	11.07	2.32	8.32
19	Non reducing sugar	mg/g	10.01	10.08	1.09	0.11	0.64
20	Fruit yield/plant	kg	13.11	13.13	36.94	4.85	5.05

GCV- Genotypic co-efficient of variation, PCV- Phenotypic co-efficient of variation, h^2- Heritability (broad sense), GA- Genetic advance, GAM- Genetic advance as % mean.

Table 2. Genotypic correlation between morphological, yield, and fruit quality characters in bael genotypes

Trait	1	2	3	4	5	6	7	8	9	10	11	12	13	14	15	16	17	18	19	20
1	**1**	0.72**	0.64**	0.07	0.59**	0.65**	0.65**	0.64**	0.50**	0.45**	0.08	-0.48	0.17*	0.08	-0.13	-0.02	-0.05	-0.12	-0.05	0.55**
2		**1**	0.99**	0.68**	0.53**	0.86**	0.86**	0.85**	0.67**	0.60**	0.06	-0.71	-0.53	0.04	0.01	0.01	-0.04	-0.10	-0.01	0.78**
3			**1**	0.74**	0.49**	0.85**	0.85**	0.84**	0.66**	0.58**	0.03	-0.72	-0.61	0.04	0.03	0.01	-0.03	-0.09	-0.01	0.77**
4				**1**	0.07	0.57**	0.57**	0.57**	0.34**	0.28**	-0.15	-0.70	-0.95	-0.01	0.21**	0.05	0.003	0.040	0.01	0.65**
5					**1**	0.33**	0.33**	0.34**	0.44**	0.34**	0.16	-0.28	0.02	0.01	-0.14	-0.03	0.03	0.03	0.10	0.31**
6						**1**	0.98**	0.92**	0.60**	0.59**	-0.02	-0.58	-0.41	0.18	-0.01	0.01	-0.06	-0.24	-0.09	0.70**
7							**1**	0.92**	0.60**	0.59**	-0.02	-0.58	-0.41	0.18	-0.01	0.01	-0.06	-0.24	-0.09	0.70**
8								**1**	0.59**	0.55**	0.01	-0.61	-0.42	0.12	0.004	0.03	0.002	-0.15	-0.004	0.67**
9									**1**	0.89**	0.06	-0.12	-0.27	0.14	-0.05	0.06	-0.09	-0.16	0.02	0.46**
10										**1**	0.10	0.03	-0.24	0.22*	-0.01	0.05	-0.17	-0.27	-0.04	0.39**
11											**1**	-0.09	0.02	-0.14	-0.15	-0.03	0.05	-0.07	0.15*	0.11
12												**1**	0.56**	0.10	-0.02	-0.003	-0.10	-0.08	-0.06	-0.73
13													**1**	0.06	-0.20	-0.04	-0.01	-0.04	-0.04	-0.52
14														**1**	0.16*	0.08	0.04	-0.06	0.03	0.01
15															**1**	0.14	-0.15	0.08	-0.21	0.09
16																**1**	0.01	0.05	-0.004	0.06
17																	**1**	0.22**	0.91**	0.01
18																		**1**	0.20*	-0.08
19																			**1**	-0.01
20																				**1**

For trait numbers, please see Table 1.

Table 3. Eigenvalues, percent variability, and cumulative percent variability for various principal components in bael genotypes

Principal components	Eigenvalue	Percentage of variance (%)	Cumulative percentage of variance (%)
PC-1	8.12	40.19	40.19
PC-2	2.32	11.49	51.68
PC-3	2.14	10.60	62.29
PC-4	1.47	7.25	69.54
PC-5	1.19	5.88	75.42
PC-6	1.08	5.36	80.78
PC-7	0.94	4.65	85.43
PC-8	0.84	4.16	89.59
PC-9	0.65	3.19	92.79
PC-10	0.56	2.77	95.56
PC-11	0.36	1.78	97.34
PC-12	0.20	0.99	98.33

Table 4. The PCA scores for bael genotypes

Sr. No	Genotypes	PC1	PC2	Sr. No	Genotypes	PC3	PC4
1	CHESB-1	77.08	-36.61	52	CHESB-52	89.98	-34.42
2	CHESB-2	22.66	-1.20	53	CHESB-53	50.59	32.06
3	CHESB-3	7.31	-12.61	54	CHESB-54	-26.69	5.32
4	CHESB-4	29.72	37.58	55	CHESB-55	-62.17	35.37
5	CHESB-5	-8.69	-19.47	56	CHESB-56	50.81	17.78
6	CHESB-6	-48.87	30.62	57	CHESB-57	-54.67	-25.26
7	CHESB-7	-43.44	-26.65	58	CHESB-58	47.47	45.10
8	CHESB-8	53.69	-41.42	59	CHESB-59	-23.37	40.76
9	CHESB-9	-61.97	4.27	60	CHESB-60	-24.77	-97.67
10	CHESB-10	-59.91	-1.64	61	CHESB-61	-115.03	-0.03
11	CHESB-11	83.54	-49.81	62	CHESB-62	43.25	12.54
12	CHESB-12	-134.50	34.76	63	CHESB-63	-14.44	-10.03
13	CHESB-13	4.74	19.38	64	CHESB-64	55.24	-4.63
14	CHESB-14	-15.86	14.97	65	CHESB-65	-5.37	18.63
15	CHESB-15	-24.24	5.29	66	CHESB-66	-0.90	10.61
16	CHESB-16	29.98	-54.71	67	CHESB-67	-123.60	15.79
17	CHESB-17	34.05	59.15	68	CHESB-68	10.04	28.14
18	CHESB-18	46.99	59.94	69	CHESB-69	30.96	-12.29
19	CHESB-19	53.48	35.09	70	CHESB-70	12.96	25.73
20	CHESB-20	44.23	37.85	71	CHESB-71	80.96	-14.92
21	CHESB-21	10.34	-35.03	72	CHESB-72	10.06	4.41
22	CHESB-22	-61.76	-46.94	73	CHESB-73	79.02	-3.31
23	CHESB-23	49.01	40.35	74	CHESB-74	-0.04	5.47
24	CHESB-24	-45.94	-14.65	75	CHESB-75	-122.72	13.41
25	CHESB-25	146.64	156.65	76	CHESB-76	5.59	25.66
26	CHESB-26	-57.52	-3.89	77	CHESB-77	45.12	7.11
27	CHESB-27	60.35	-61.91	78	CHESB-78	-0.14	-13.00
28	CHESB-28	-116.87	3.03	79	CHESB-79	90.63	-34.41
29	CHESB-29	129.25	-22.11	80	CHESB-80	-51.48	-33.89
30	CHESB-30	-54.21	-5.76	81	CHESB-81	53.18	19.00
31	CHESB-31	4.82	1.31	82	CHESB-82	-47.50	-32.55
32	CHESB-32	-39.86	-35.34	83	CHESB-83	74.49	1.06
33	CHESB-33	-8.55	-10.02	84	CHESB-84	-46.70	1.74
34	CHESB-34	82.30	-14.10	85	CHESB-85	82.49	-35.89
35	CHESB-35	-56.28	2.84	86	CHESB-86	-56.56	-11.43
36	CHESB-36	-121.50	11.67	87	CHESB-87	-125.27	39.87
37	CHESB-37	25.46	45.29	88	CHESB-88	39.57	16.28
38	CHESB-38	-36.12	-19.41	89	CHESB-89	15.00	6.65
39	CHESB-39	99.23	-42.74	90	CHESB-90	-116.87	9.44
40	CHESB-40	-55.09	-25.13	91	CHESB-91	26.11	41.30
41	CHESB-41	87.89	-29.96	92	CHESB-92	-56.48	-25.89
42	CHESB-42	-21.84	-2.67	93	CHESB-93	56.23	33.21
43	CHESB-43	37.77	42.94	94	CHESB-94	-51.00	-5.73
44	CHESB-44	-54.90	-30.14	95	CHESB-95	84.57	-28.59
45	CHESB-45	99.05	-43.59	96	CHESB-96	6.12	16.57
46	CHESB-46	-22.25	-6.54	97	CHESB-97	-38.90	0.35
47	CHESB-47	-24.80	8.49	98	CHESB-98	85.92	-19.23
48	CHESB-48	-51.41	-31.20	99	CHESB-99	-64.73	-12.58
49	CHESB-49	11.84	-4.11	100	CHESB-100	-101.79	18.31
50	CHESB-50	69.69	-33.59	101	CHESB-101	-65.97	-8.58
51	CHESB-51	-3.94	28.12				

Table 5. Cluster composition based on D^2 statistics of bael genotypes in six clusters

Cluster	No. of Genotype	Genotypes
Cluster I	12	CHESB-2, CHESB-9, CHESB-10, CHESB-14, CHESB-26, CHESB-31, CHESB-33, CHESB-42, CHESB-48, CHESB-59, CHESB-64, CHESB-69
Cluster II	24	CHESB-7, CHESB-22, CHESB-24, CHESB-30, CHESB-32, CHESB-35, CHESB-38, CHESB-40, CHESB-44, CHESB-47, CHESB-54, CHESB-55, CHESB-57, CHESB-60, CHESB-80, CHESB-82, CHESB-84, CHESB-86, CHESB-89, CHESB-92, CHESB-94, CHESB-97, CHESB-99, CHESB-101
Cluster III	19	CHESB-3, CHESB-18, CHESB-23, CHESB-29, CHESB-34, CHESB-39, CHESB-41, CHESB-45, CHESB-50, CHESB-52, CHESB-71, CHESB-73, CHESB-79, CHESB-81, CHESB-83, CHESB-85, CHESB-93, CHESB-95, CHESB-98
Cluster IV	09	CHESB-12, CHESB-28, CHESB-36, CHESB-61, CHESB-67, CHESB-75, CHESB-87, CHESB-90, CHESB-100
Cluster V	36	CHESB-1, CHESB-4, CHESB-5, CHESB-6,CHESB-8, CHESB-11, CHESB-13, CHESB-15, CHESB-16, CHESB-17, CHESB-19, CHESB-20, CHESB-21 , CHESB-25, CHESB-27, CHESB-37, CHESB-43, CHESB-46, CHESB-49, CHESB-53, CHESB-56, CHESB-58, CHESB-62, CHESB-63, CHESB-65, CHESB-66, CHESB-68, CHESB-70, CHESB-72, CHESB-74, CHESB-76, CHESB-77, CHESB-78, CHESB-88, CHESB-91, CHESB-96
Cluster VI	01	CHESB-51

Table 6. Cluster mean analysis for 20 traits in beal genotypes

S.N	Character	Clusters					
		I	II	III	IV	V	VI
1	Shell weight	222.51	256.37	149.42	304.51	179.68	198.13
2	Fruit weight	1.30	1.62	0.84	2.48	1.49	1.50
3	Pulp weight	1.00	1.29	0.63	2.10	1.24	1.23
4	Pulp	76.74	79.57	74.46	84.69	83.12	82.29
5	Shell thickness	2.29	3.03	2.43	3.37	2.34	2.42
6	Fruit circumference	43.36	42.09	30.49	54.18	41.17	42.00
7	Fruit width	13.80	13.39	9.70	17.24	13.10	13.36
8	Fruit length	15.53	15.58	11.72	19.51	15.42	15.84
9	No. of seeds/fruit	148.20	148.64	96.65	211.74	138.69	165.98
10	Total seed weight	29.44	27.02	22.18	34.14	26.62	29.15
11	Fiber weight	46.82	44.94	39.80	40.30	42.33	38.34
12	Seed	2.31	1.68	2.72	1.38	1.81	1.94
13	Shell	17.26	15.96	18.06	12.30	12.18	13.21
14	TSS Pulp	40.20	38.03	37.65	38.79	37.90	40.55
15	Vitamin C	17.99	17.46	18.63	18.19	19.75	23.35
16	Acidity	0.55	0.58	0.57	0.54	0.54	0.56
17	Total sugar	20.30	20.93	20.67	20.13	20.08	20.65
18	Phenolic content	20.25	28.33	29.95	26.19	29.07	30.62
19	Non Reducing sugar	17.04	17.77	17.53	17.04	16.68	17.16
20	Fruit yield/plant	93.34	103.96	75.45	108.99	99.73	104.56

Table 7. Values of partial regression coefficient for growth and quality traits in beal genotypes

S.N	Character	Coef	Std error	t	P> t	[0.025	0.975]
1	Shell weight	-248.213	236.948	-1.048	0.298	-719.578	223.152
2	Fruit weight	0.012	0.094	0.129	0.898	-0.175	0.199
3	Pulp weight	-0.483	5.066	-0.095	0.924	-10.561	9.595
4	Pulp	-0.484	5.066	-0.096	0.924	-10.562	9.594
5	Shell thickness	3.474	2.346	1.480	0.143	-1.194	8.141
6	Fruit circumference	-1.281	1.931	-0.663	0.509	-5.122	2.561
7	Fruit width	-158.811	204.163	-0.778	0.439	-564.956	247.333
8	Fruit length	499.991	641.462	-0.779	0.438	-776.080	1776.062
9	No. of seeds/fruit	-1.043	0.828	-1.260	0.211	-2.690	0.604
10	Total seed weight	0.031	0.043	0.717	0.476	-0.055	0.118
11	Fiber weight	0.579	0.602	0.963	0.338	-0.617	1.776
12	Seed	0.306	0.179	1.716	0.090	-0.049	0.662
13	Shell	-7.538	5.511	-1.368	0.175	-18.502	3.425
14	TSS pulp	3.387	3.437	0.985	0.327	-3.450	10.224
15	Vitamin C	-0.169	0.273	-0.618	0.538	-0.712	0.374
16	Acidity	0.215	0.246	0.872	0.386	-0.275	0.704
17	Total sugar	0.075	0.130	0.582	0.562	-0.182	0.333
18	Phenolic content	1.866	1.209	1.543	0.127	-0.540	4.271
19	Non reducing sugar	-0.069	0.137	-0.505	0.615	-0.314	0.203
20	Fruit yield/plant	-1.690	1.161	-1.456	0.149	-3.999	0.619

Dep. variable: fruit yield/plant (kg)	Log-Likelihood:-329.31
R-squared: 0.749	Adj. R-squared: 0.694
F-statistic: 13.58	Prob (F-statistic): 1.20e-17
AIC: 696.6	BIC: 746.3
Omnibus: 1.715	Prob (Omnibus): 0.424
Durbin-Watson: 1.928	Cond. No: 371e+18
Jarque-Bera(JB): 1.229	Prob(JB): 0.541
Skew: -0.051	Kurtosis: 3.531

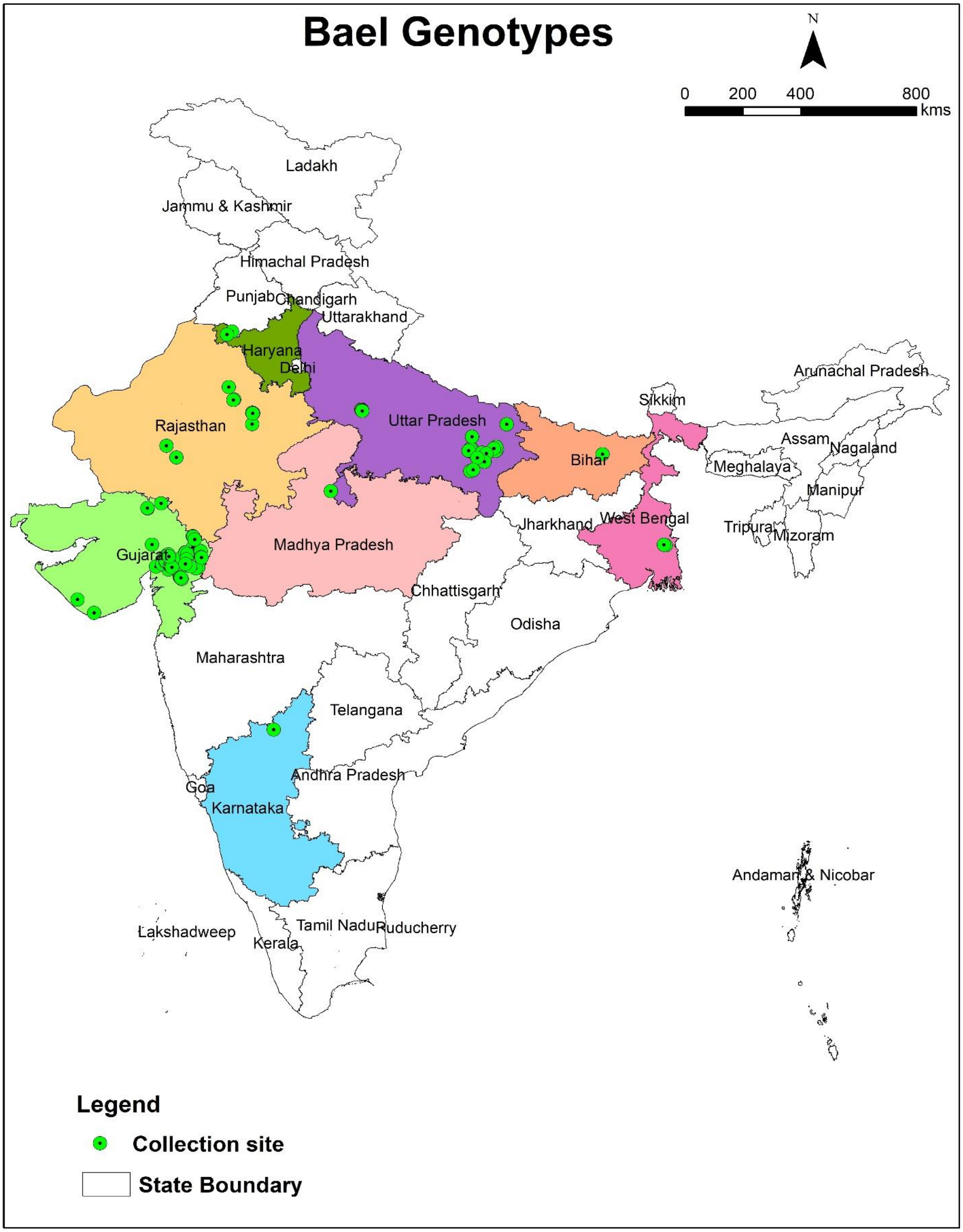

Fig. 1. Collection sites of bael genotypes

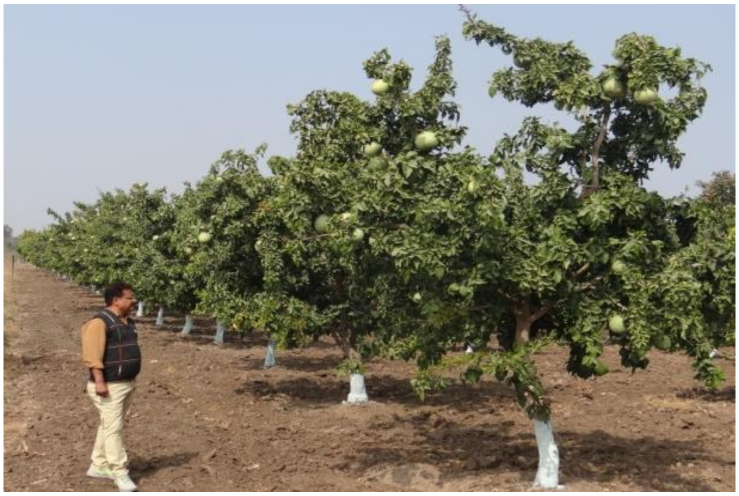

a

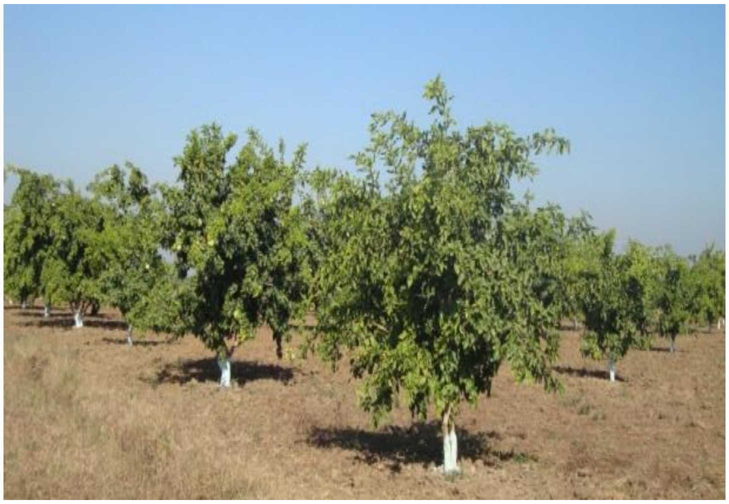

b

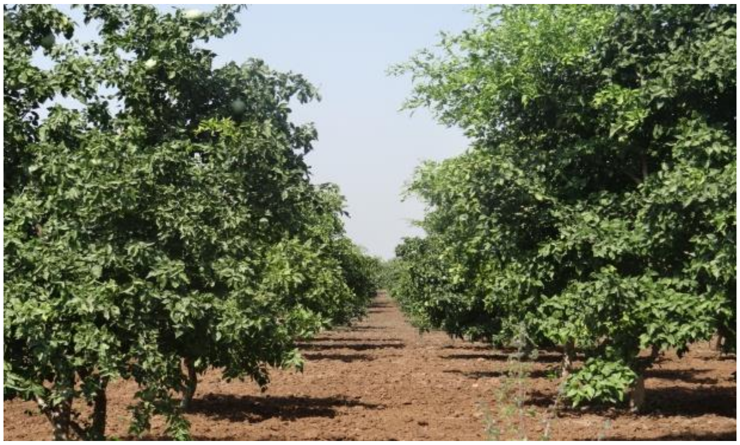

c

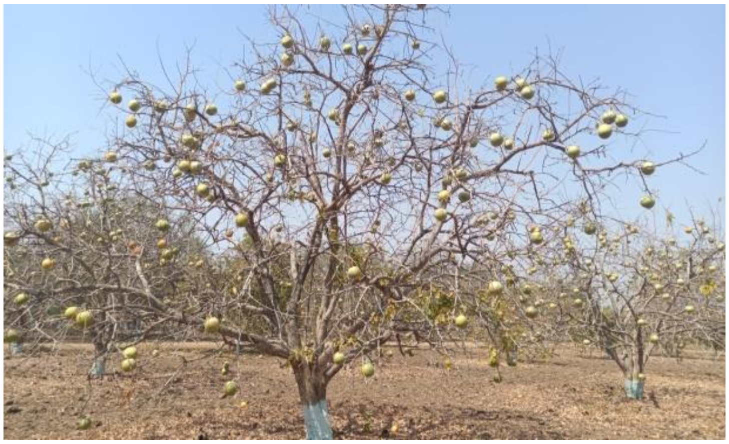

d

Fig. 2. Different bael genotypes at field repository of ICAR-CHES, Godhra, Gujarat, India

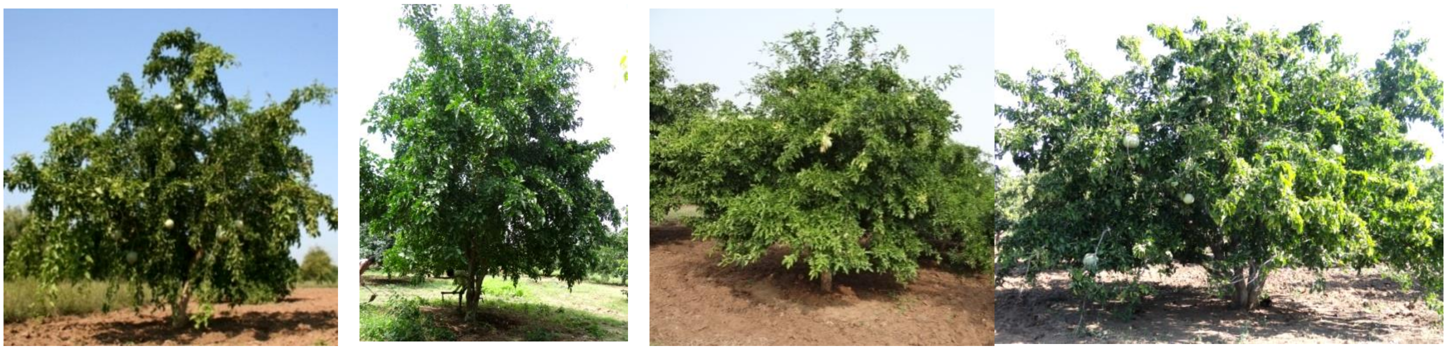

a b c d

Fig 3. Growth behavior of bael genotypes: a) drooping, b) erect, c) semi-spreading, and d) spreading

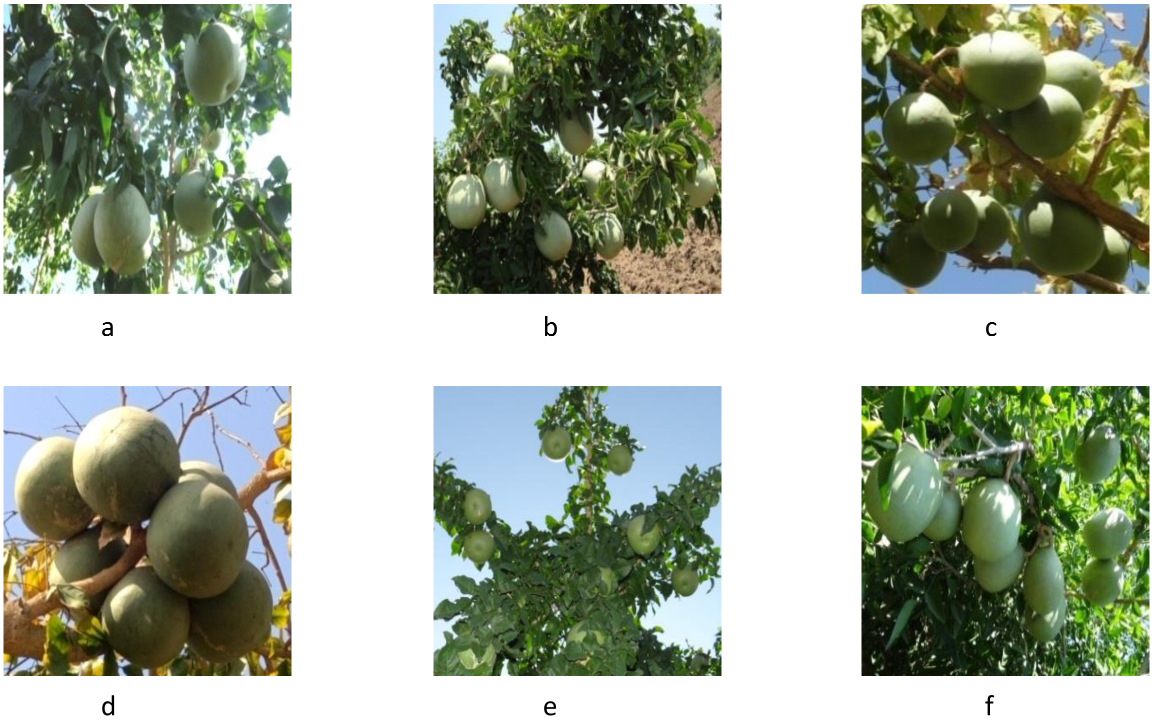

Fig. 4. Bearing behavior variation in fruit of different bael genotypes: a) CHESB-11, b) CHESB-1, c) CHESB-8, d) CHESB-27, e) CHESB-31, and f) CHESB-16

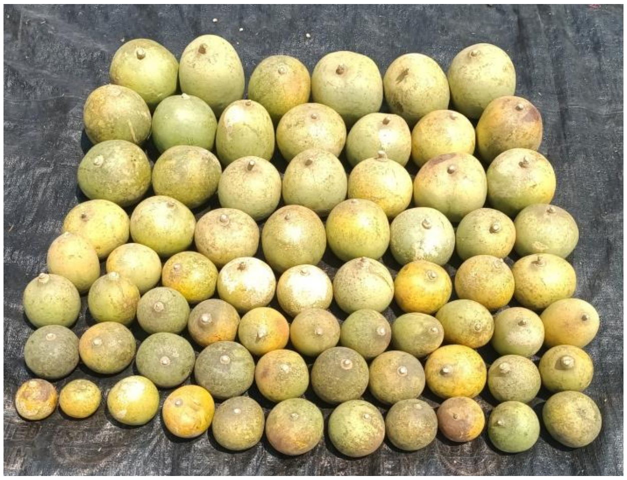

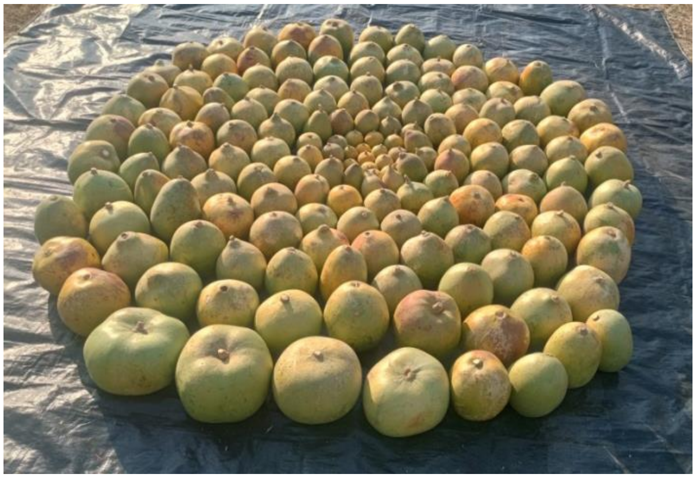

Fig. 5. Variability in fruit shape and size of bael genotypes.

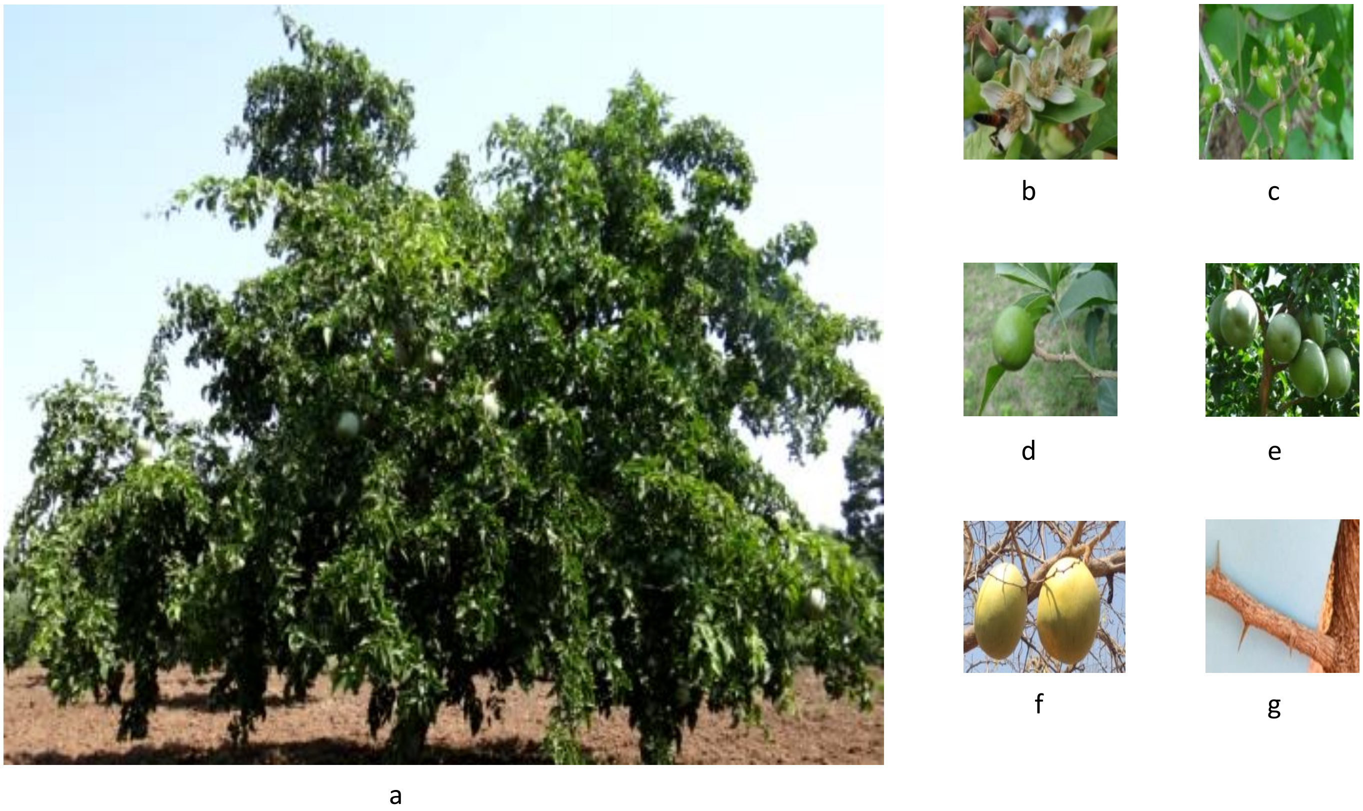

Fig. 6. Representative photographs of bael genotype conserved at field gene bank ICAR-CIAH-CHES, Godhra, Gujarat: a) full-grown tree, b) flower and pollinator, c) fruit set, d) developing fruit, e) full-developed fruit, f) ripened fruit, and g) thorn size

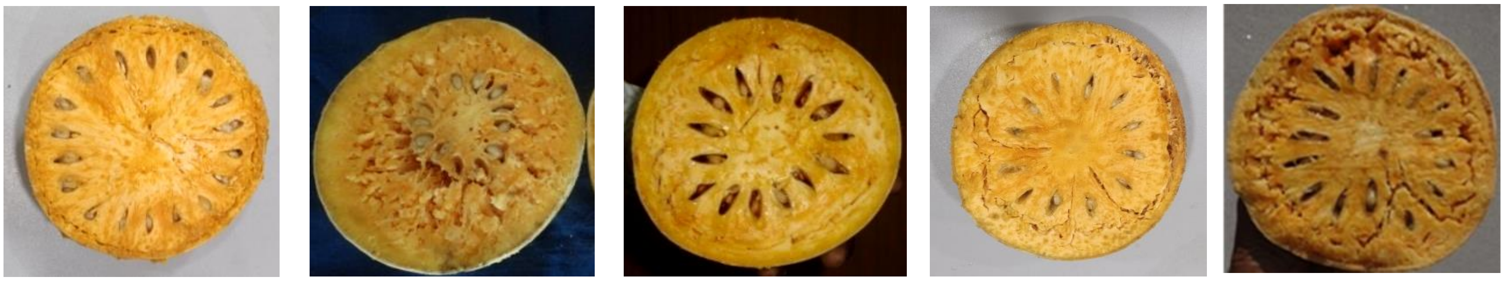

a b c d e

Fig. 7. Variation in locule arrangement of different bael genotypes: a) peripheral, b) highly centric, c) centric, d) semi-centric, and e) scattered

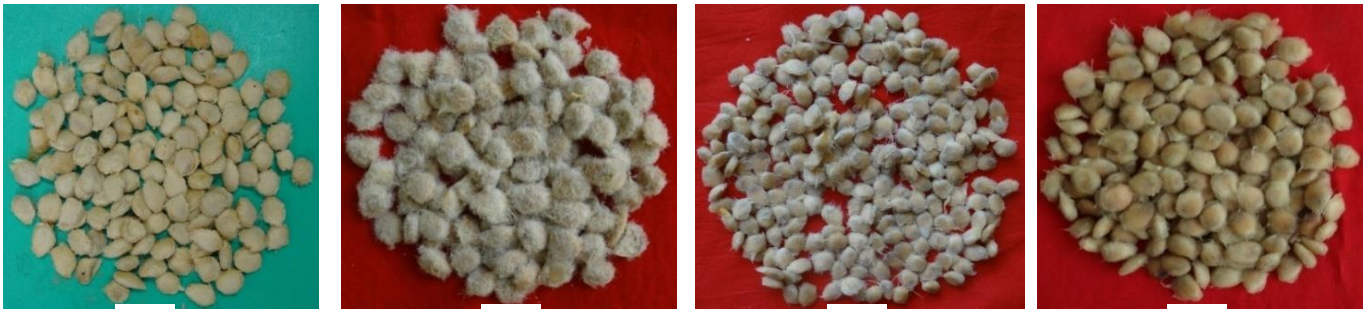

a b c d

Fig. 8. Variation in seed size, shape, and fiber of different bael genotypes: a) small seed and fiberless, b) bold seed with fiber, c) small seed with fiber, and d) bold seed and fiberless

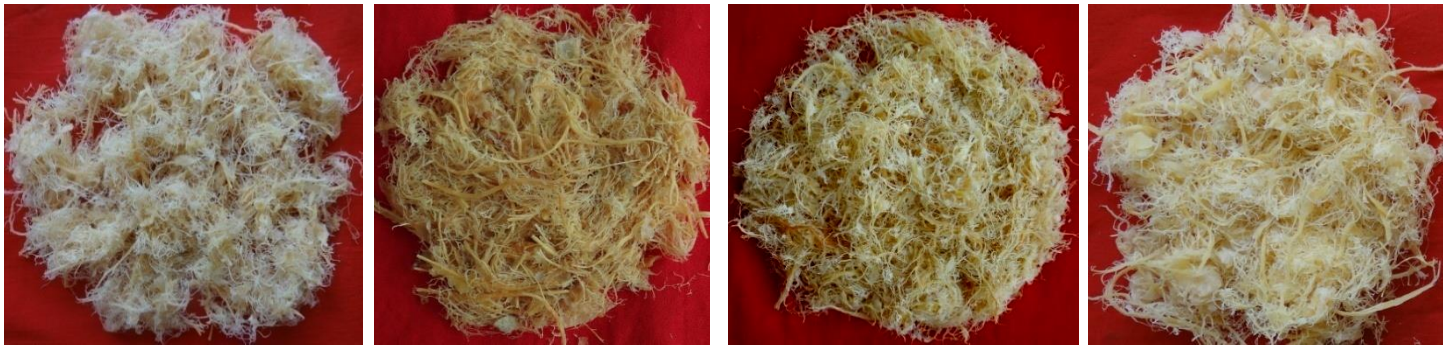

a b c d

Fig. 9. Variation in fiber color of different bael genotypes: a) whitish-yellow, b) yellow, c) light yellow, and d) yellowish-white

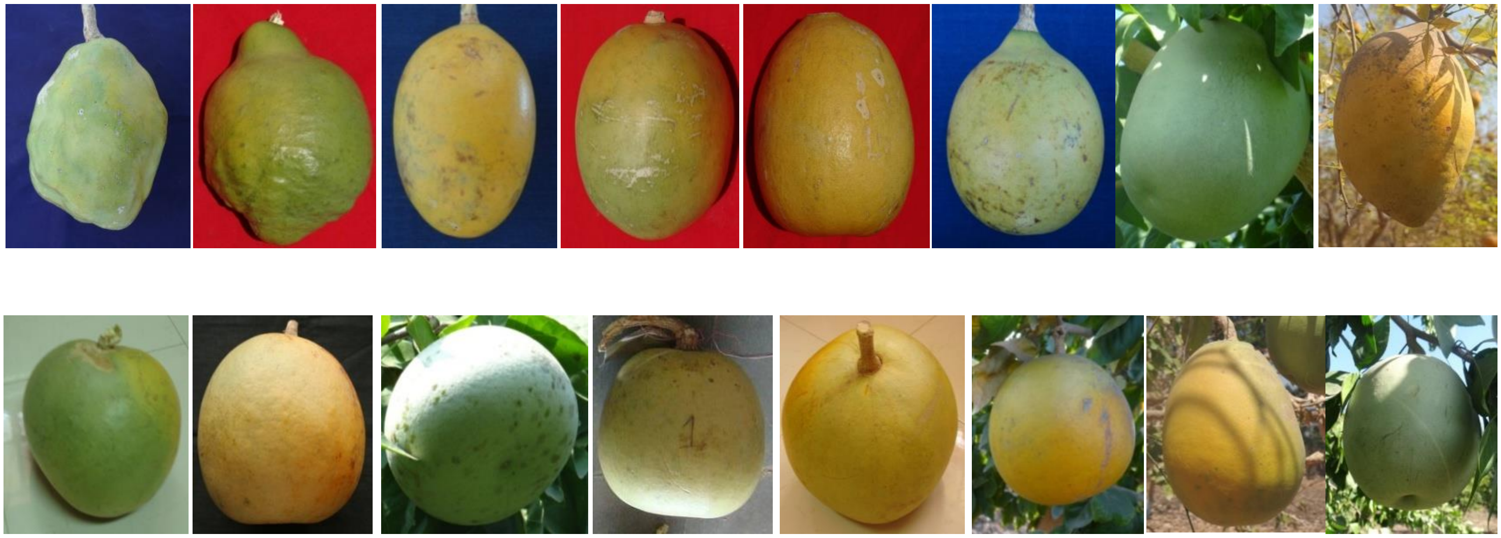

Fig. 10. Variation in fruit size and shape of different bael genotypes under rainfed semi-arid conditions

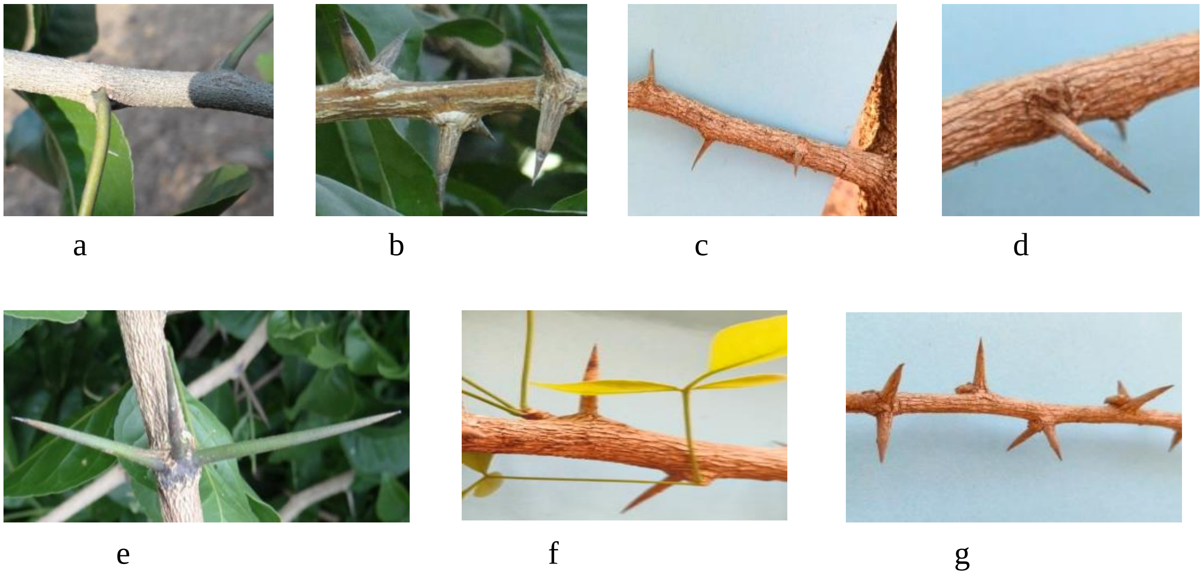

Fig. 11. Variation in thorn orientation size and shape of different bael genotypes: a) thornless, b) double at node, medium, and stout, c) single, small, and thin, d) single, long, and stout, e) three at node, long, and medium thin, f) single at node, medium, and stout, and g) double at node, small, bold, and stout

a b c d

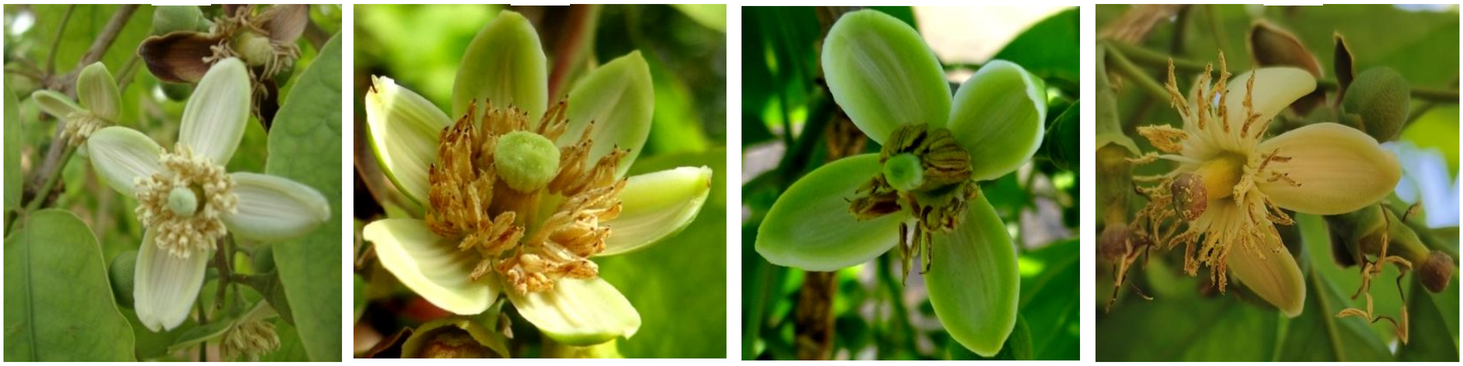

Fig. 12. Variation in stigma color in bael genotypes: a) greenish-white, b) light green, c) green, and d) purple

a b c

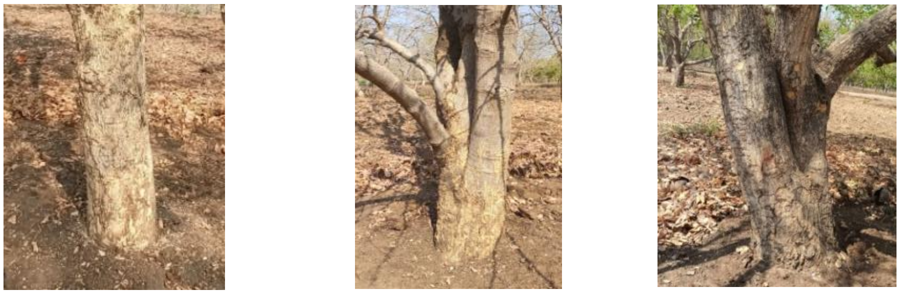

Fig. 13. Variation in stem bark color of bael genotypes: a) grey-brown, b) yellow-grey, and c) brown-grey

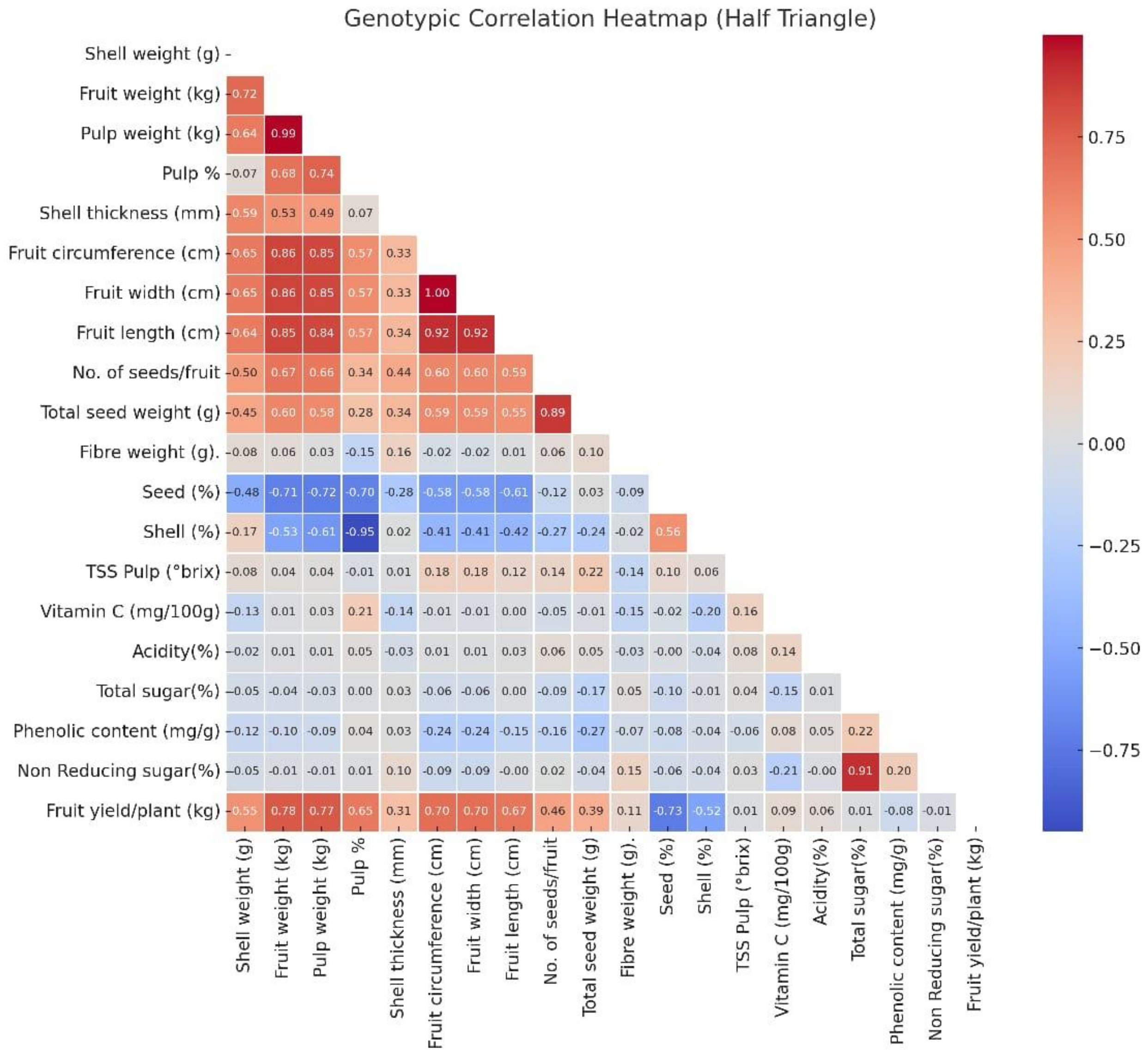

Fig. 14. Genotypic correlation coefficient heatmap between 20 yield and quality characters in bael genotypes.

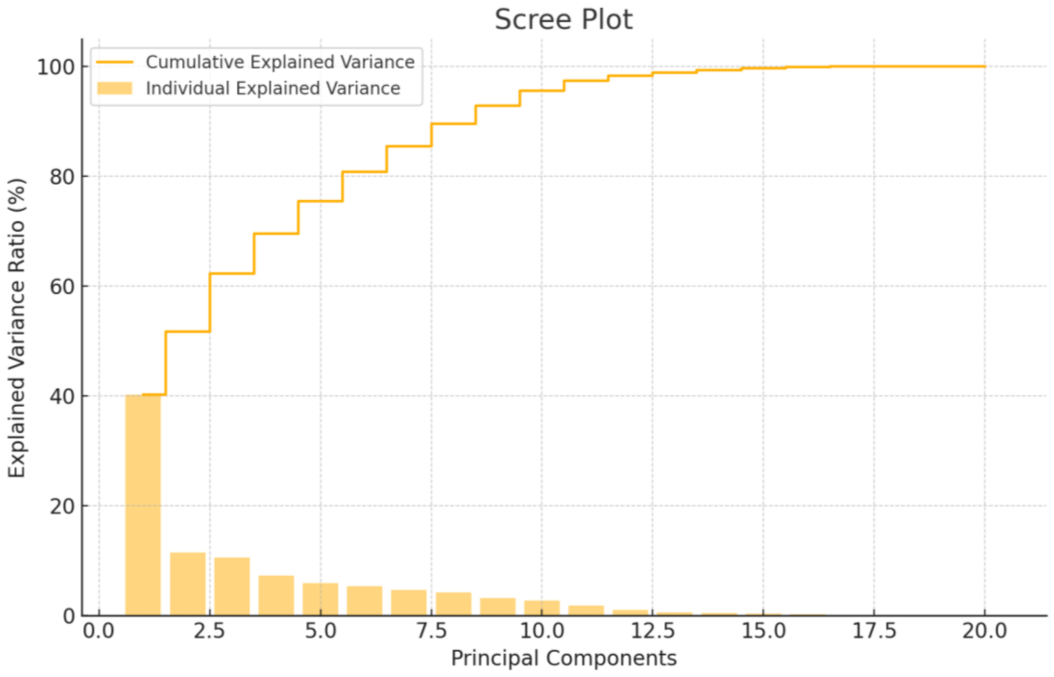

Fig. 15. PCA scree plot of bael genotypes between explained variance ratio and principal components

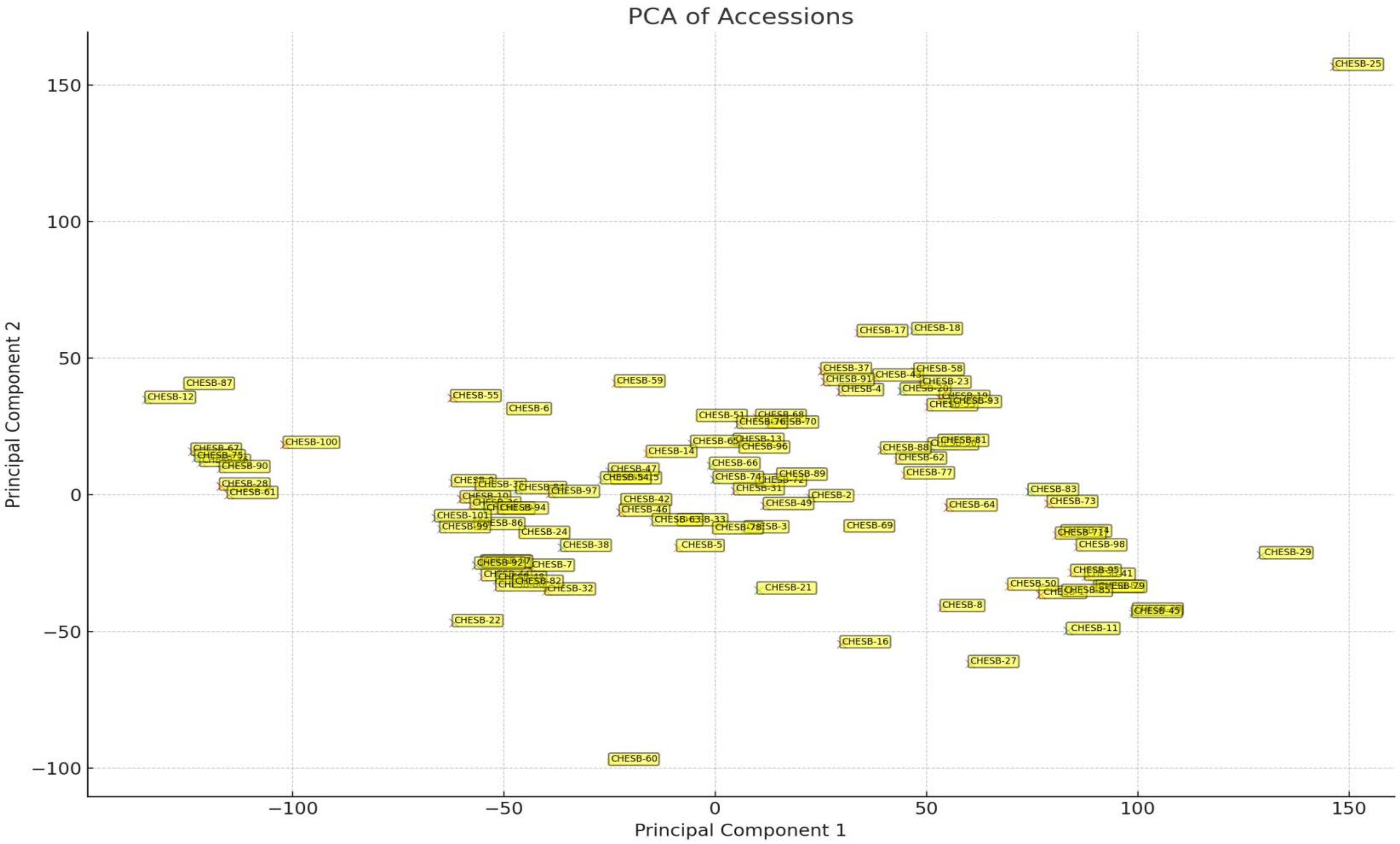

Fig. 16. Distributions of bael genotypes across two PCs

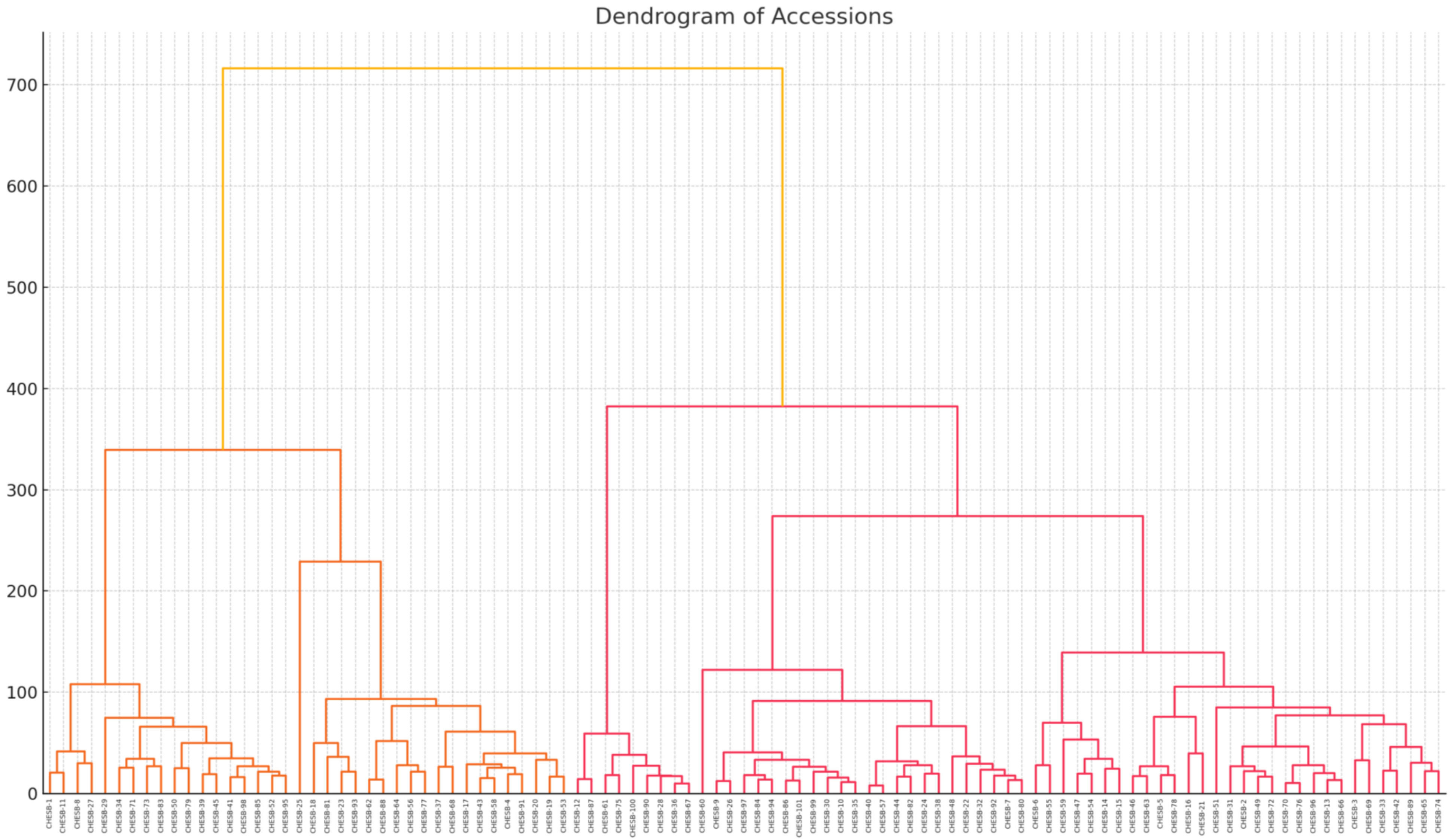

Fig. 17. Dendrogram showing the relationship among bael genotypes

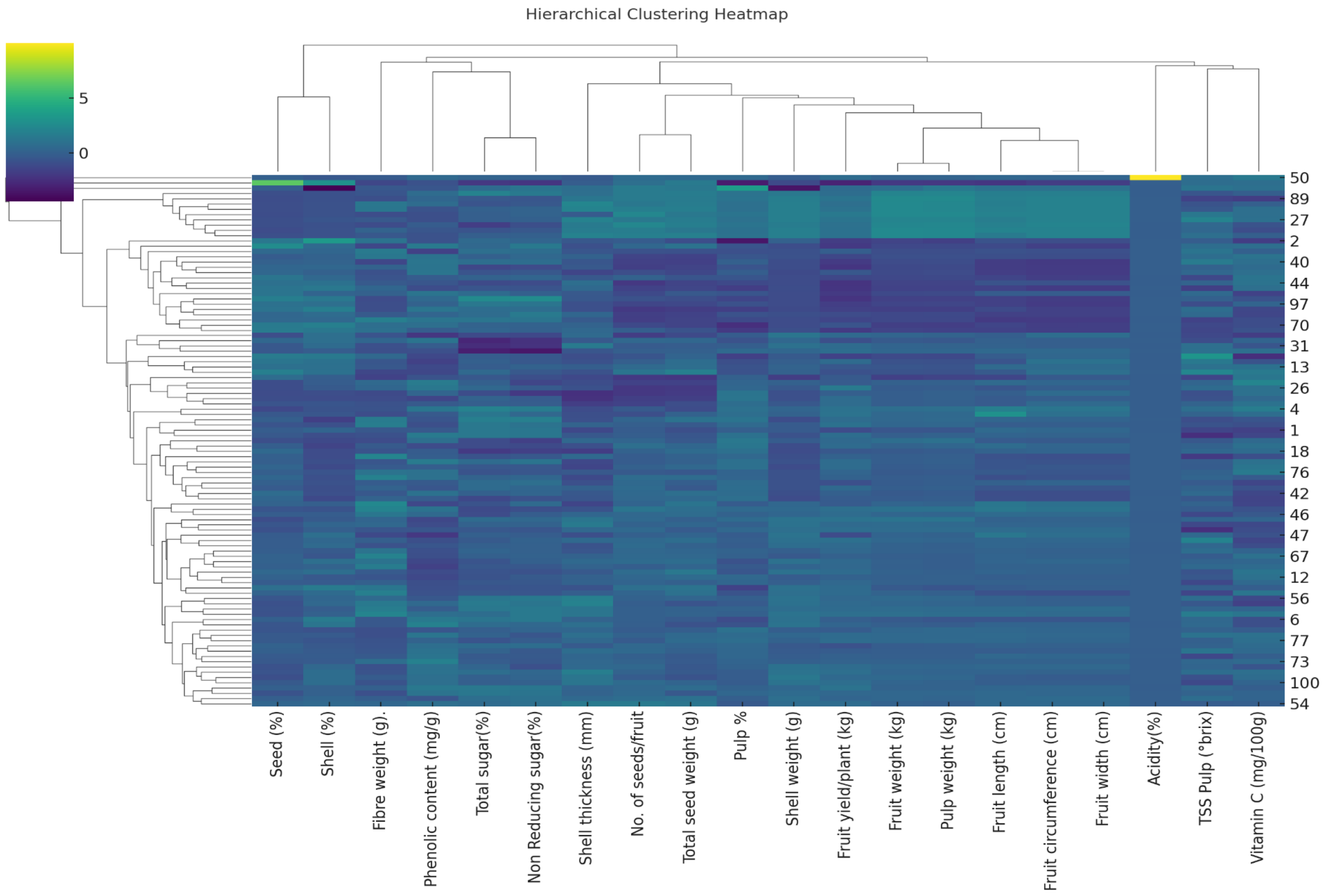

Fig. 18. Hierarchical clustering heatmap (HCH) represents 20 yield and quality characters of bael genotype

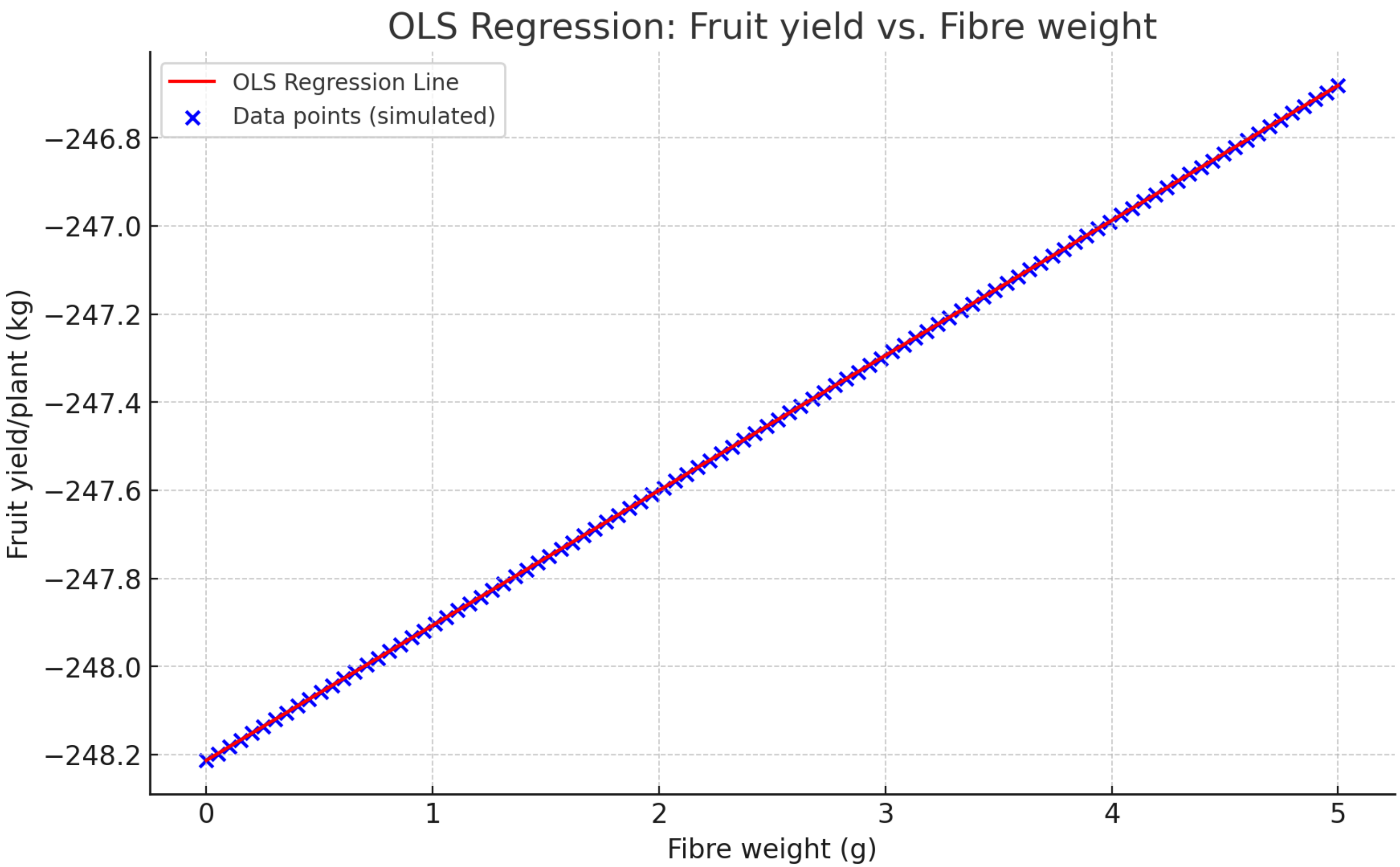

Fig. 19. The association between fiber weight (g) and fruit yield/plant (kg) based on the OLS regression model in bael genotypes.
