# Supplementary material for: Phenotypic variability and trait-specific selection in Aegle marmelos Correa genotypes based on morphological and quality traits
Source: PLoS One. 2026 Apr 20;21(4):e0347746. doi: 10.1371/journal.pone.0347746 (PMC13095130; doi:10.1371/journal.pone.0347746)
Supplement: Attachment — Submitted filename: Response.docx [file pone.0347746.s004.docx]

**Subject:** Resubmission of Revised Manuscript (PONE-D-26-04072R1)

Dear Dr. Ramos,

Thank you for your message and for checking our submission.

We have carefully followed your instructions and uploaded all figures as separate high-resolution files, in accordance with PLOS ONE guidelines. The manuscript has been revised accordingly and resubmitted through the system.

We kindly ask you to proceed with the review process.

Thank you for your time and consideration.

Kind regards,
Prof. Dr. Ali Khadivi
Corresponding author
(On behalf of all authors)
